# Estimating the Hidden Burden of Bovine Tuberculosis in Great Britain

**DOI:** 10.1371/journal.pcbi.1002730

**Published:** 2012-10-18

**Authors:** Andrew J. K. Conlan, Trevelyan J. McKinley, Katerina Karolemeas, Ellen Brooks Pollock, Anthony V. Goodchild, Andrew P. Mitchell, Colin P. D. Birch, Richard S. Clifton-Hadley, James L. N. Wood

**Affiliations:** 1 Disease Dynamics Unit (DDU), Department of Veterinary Medicine, University of Cambridge, Cambridge, United Kingdom; 2 Animal Health and Veterinary Laboratories Agency Weybridge, New Haw, Addlestone, United Kingdom; Imperial College London, United Kingdom

## Abstract

The number of cattle herds placed under movement restrictions in Great Britain (GB) due to the suspected presence of bovine tuberculosis (bTB) has progressively increased over the past 25 years despite an intensive and costly test-and-slaughter control program. Around 38% of herds that clear movement restrictions experience a recurrent incident (breakdown) within 24 months, suggesting that infection may be persisting within herds. Reactivity to tuberculin, the basis of diagnostic testing, is dependent on the time from infection. Thus, testing efficiency varies between outbreaks, depending on weight of transmission and cannot be directly estimated. In this paper, we use Approximate Bayesian Computation (ABC) to parameterize two within-herd transmission models within a rigorous inferential framework. Previous within-herd models of bTB have relied on ad-hoc methods of parameterization and used a single model structure (SORI) where animals are assumed to become detectable by testing before they become infectious. We study such a conventional within-herd model of bTB and an alternative model, motivated by recent animal challenge studies, where there is no period of epidemiological latency before animals become infectious (SOR). Under both models we estimate that cattle-to-cattle transmission rates are non-linearly density dependent. The basic reproductive ratio for our conventional within-herd model, estimated for scenarios with no statutory controls, increases from 1.5 (0.26–4.9; 95% CI) in a herd of 30 cattle up to 4.9 (0.99–14.0) in a herd of 400. Under this model we estimate that 50% (33–67) of recurrent breakdowns in Britain can be attributed to infection missed by tuberculin testing. However this figure falls to 24% (11–42) of recurrent breakdowns under our alternative model. Under both models the estimated extrinsic force of infection increases with the burden of missed infection. Hence, improved herd-level testing is unlikely to reduce recurrence unless this extrinsic infectious pressure is simultaneously addressed.

## Author Summary

Epidemic models are commonly used to assess the impact of alternative management strategies. The efficacy of controls is typically assumed from “expert opinion” rather than estimated from data. Managed endemic diseases such as bovine tuberculosis offer the potential to estimate the efficiency of control directly from epidemiological data. Our methodology constitutes a shift in the level of statistical rigor applied to “policy” models and offers insights into the epidemiology of Bovine tuberculosis in Great Britain. bTB continues to persist and spread relentlessly in Britain, despite extensive testing and control programs. Cattle farmers question the efficacy of cattle controls, blaming the badger wildlife reservoir. Contrary to much public perception, we demonstrate the importance of cattle-to-cattle transmission, especially in larger herds. We estimate that in the worst case scenario up to 21% of herds may be harboring infection after they clear restrictions. However, we also estimate that there is a high rate of re-introduction of infection into herds, particularly in high incidence areas. Eliminating the hidden burden of infection alone is unlikely to be sufficient to prevent recurrent breakdowns. Rather, the high rate of external infection, both through cattle movements and environmental sources, must be addressed if recurrence is to be reduced.

## Introduction

The number of cattle herds in Great Britain (GB) placed under movement restrictions due to the suspected presence of bovine tuberculosis (bTB) has progressively increased over the past 25 years [1]. This increase in the rate of so-called “breakdown” herds is despite an intensive and costly test-and-slaughter control program [2]. Recent studies have focused on estimating the contributions of cattle movements and wildlife transmission to incidence [1],[3]–[8] as measured by the rate of new breakdowns. However, less attention has been paid to quantifying the dynamics of transmission within herds, even though this is arguably the most data-rich unit within the wider ecology of *M. bovis*. Previous history of disease within a herd is an important predictor of breakdown [5], [8], [9], with 38% of herds that clear movement restrictions experiencing a recurrent incident within 24 months [10].

This high rate of recurrence suggests that infection may be persisting within herds in the face of repeated testing. In GB and internationally, detection and clearance of herds is dependent on variants of the imperfect tuberculin skin test. In GB and Ireland this takes the form of a single intra-dermal comparative cervical tuberculin (SICCT) [11] test. Infection missed by SICCT testing is likely to be contributing to recurrence within herds. However, the lack of a gold-standard diagnostic test for bovine tuberculosis means that the efficiency of the SICCT test is poorly characterized and the contribution of missing infection to recurrence cannot be easily assessed. Furthermore, reactivity to the SICCT test is dependent on the time-from-infection [12], most often characterized [13], [14] as an *occult period* where animals are infected but not yet detectable. As a consequence, the efficiency of testing within a herd depends not only on the characteristics of the diagnostic test, but also on the competing timescales of transmission and the frequency of testing.

Within-herd models of bTB have been developed to address this problem with a view to informing government policy [13], [14]. However, extant models have been based on ad-hoc parameterizations informed by disparate experimental studies and expert opinion. There exists considerable uncertainty in the assumed values of key parameters, in particular the occult period, the scaling of transmission rates with herd size [15], [16] and the duration of latency between infection and infectiousness. To address this uncertainty, we propose a novel basis for parameterization of within-herd models using measures of stochastic persistence [17] as a metric for Approximate Bayesian Computation (ABC) [18]–[19].

Persistence measures have proven to be a powerful probe on which to parameterize models of childhood infectious diseases [20]–[23]. A successful approach has been to assume that the intrinsic rate of transmission within a population is rapid compared to the combined rate of transmission from sources extrinsic to the local population. This time-scale separation allows us to model local populations independently. Extrinsic routes of transmission are modeled through a generalized infectious pressure [20]–[22]. Comparatively less theoretical attention has been paid to modeling the persistence of managed endemic diseases.

For chronic diseases, such as bTB, demographic turnover of the population is the only natural mechanism acting to clear infection from populations. In this context persistence can be used as an indirect measure of the efficiency of diagnostic testing. In this study we model the within-herd persistence of bTB as a balance between three key processes: the infectious pressure acting to introduce infection into the herd from extrinsic sources, the rate of cattle-to-cattle transmission within the herd and the rate of removal of infection through testing and demographic turnover. Herds are considered as isolated populations loosely connected to a reservoir of infection modeled as an infectious pressure. We are therefore not concerned with modeling the routes of introduction to the herd – which may be through movements of infected animals or contact with wildlife reservoir populations. Instead we focus on the processes of transmission within a herd with relation to the detection and resolution of breakdowns. We do so using two mechanistic models of within-herd transmission that we parameterize using routinely collected epidemiological data. We finally apply our parameterized models to estimate the hidden burden of infection and its implications for control of bTB in Great Britain.

## Results

### Measures of persistence of bTB

The probability of extinction within epidemic models is dependent on the past history of infection within the population [24]. Alternative empirical measures of persistence, that capture different aspects of the transmission dynamics, can be constructed depending on how we condition on the past history of infection [21]. For bTB, infection missed during a given test is likely to contribute to the probability of the herd failing subsequent tests. Contingent on the natural time-scale of transmission and the scheduling of testing, missing infection may act to prolong the duration of breakdowns and/or increase the probability of a recurrent breakdown. We therefore quantify within-herd persistence through two competing measures related to the duration and the rate of recurrence of breakdowns. The duration of breakdowns is captured by the probability that breakdowns are prolonged [25], defined as lasting longer than 240 days. Recurrence is captured through the probability of a breakdown recurring [10] within a fixed time horizon of 6, 12 and 24 months after the end of a breakdown.

### Study population

Bovine tuberculosis is a statutory infectious disease. Incidence and testing data are routinely collected by the Animal Health and Veterinary Laboratories Agency (AHVLA) and collated within the *VetNet* database. It is not feasible or desirable to model the full complexity of the British testing regime within a herd level model. In addition to the schedule of statutory surveillance testing, there exists a diverse range of auxiliary tests including pre-movement testing, contiguous tests and tests trigged by epidemiological investigations of “at risk” premises. As a consequence there is considerable variability in the duration of time that infection can spread unobserved within herds before detection. In an attempt to control for this uncertainty we restrict our current analysis to new breakdowns with start dates between 2003–2005 that were detected through routine surveillance, either through the slaughterhouse or by the detection of reactors at a routine or whole herd test (tests classified as ‘VE-WHT’, ‘VE-WHT2’,‘VE-RHT’ or ‘VE-SLH’).

We choose to restrict our study to the period 2003–2005 due to systematic changes to the testing system surrounding the 2001 foot-and-mouth disease epidemic [5] and a later increase in use of the gamma-interferon test [26]. Although discretionary use of the gamma interferon test increased after the end of our study period, this does not appear to have impacted upon persistence and our model still provides an equally good fit to target measures taken from more recent data (Figures **S7**,**S11**).

The cessation of testing during the 2001 foot-and-mouth epidemic artificially increased the duration of time that herds were kept under movement restrictions, delayed the scheduling of routine surveillance tests and was associated with an increase in incidence and spread of bTB to new areas [5]. Given the slow rate of transmission of bTB, the perturbative effect of this disruption to testing is likely to have continued for many years. However, our interest is primarily in the sequence of transmission and testing after the disclosure of a breakdown. Disruptions to testing prior to the disclosure of infection will increase the duration of time that infection was able to transmit silently within our study herds. However, as this variation is included within the empirical testing distributions used within our model (Figure **S1**, Dataset **S1**) we do not expect it to affect our results.

**Figure 1:**
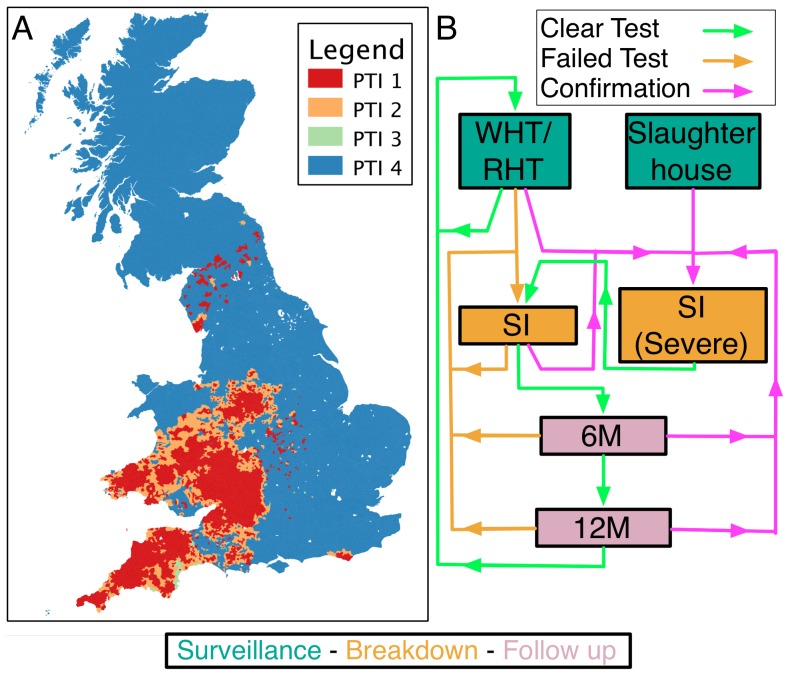
Parish Testing Intervals (PTI) for bTB in GB (2003–2005). Routine surveillance for bTB in GB is based upon the regular (SICCT) testing of herds at a frequency determined by the local incidence of affected premises. Panel (A) maps the shortest recorded PTI for each parish over our study period of 2003–2005. High incidence areas are spatially clustered with the greatest incidence, and thus intensity of testing, in the south-west of England and south Wales. PTI therefore offers a crude categorization of herds according to epidemiological risk, past history of testing and to a lesser extent, geographical location. In contrast to surveillance testing, the sequence of tests (B) following a breakdown are dependent only on the outcome of tests on the affected premises. A failed surveillance test leads to a sequence of short interval tests (SI) at intervals of at least 60 days. Confirmation of infection, through isolation of *M. bovis* or evidence of visible lesions at slaughter, leads to tests being re-interpreted under a “severe” interpretation of the SICCT, until one test is passed; testing then continues until an additional test at the standard interpretation is passed. After a breakdown is cleared follow-up tests are scheduled at intervals of at least 6 and 12 months, after which testing frequency reverts to the local parish testing frequency.

Of 10,174 breakdowns recorded within our study period, 3,456 (34%) breakdowns match our criteria for inclusion. Restricting our analyses to this sub-population has an important advantage. The scheduling of surveillance tests in GB is based on the local incidence (Figure **1**), that determines the so-called parish testing interval (PTI). The duration of time that infection may have remained undisclosed within our sub-population of herds is strongly constrained by the herd's local PTI. After detection of infection, regardless of the disclosing test type, all herds must undergo the same statutory sequence of testing. We therefore do not believe that our inclusion criteria impacts upon the generality of our inference for rates of within-herd transmission and the efficiency of surveillance.

Of the remaining breakdowns the majority are either recurrent breakdowns (2,102; 21%) initiated by a follow-up ‘VE-6M’ or ‘VE-12M’ test or breakdowns that started with a so-called “inconclusive” reactor (2,032; 20%). Inconclusive reactors (IRs) demonstrate a response to the SICCT that is close to the cut-off value defining a reactor. IRs do not necessarily trigger a breakdown but require the animal, rather than the herd, to be retested at an interval of 60 days. The population of IRs will be composed of both false reactor and truly infected animals and cannot be rationally treated within our model framework, requiring us to omit these herds from our analysis. The remaining 25% of breakdowns were initiated through a mixture of contiguous testing of affected premises and contact tracing.

The persistence of bTB has previously been demonstrated to scale with herd size [27]; a known risk factor for both prolongation [25] of breakdowns in GB and recurrence in Irish herds [28]. We extend these analyses to quantify the relationship of our two persistence measures with herd size. The size of a cattle herd varies dynamically, even over the course of a breakdown. We therefore define herd size as the maximum herd size over the breakdown. The distribution of herd sizes is right skewed with 90% of breakdowns having herd sizes less than or equal to 360 cattle (Figure **S4**). The scarcity of herd sizes larger than this limits our ability to measure the relationship with persistence [27]. We therefore finally restrict our study population to breakdowns with a herd size of less than or equal to 360 cattle, leaving us with a final study population of 3,094 breakdowns. These 3,094 breakdowns were then binned into 6 groups with histogram mid-points of [30,90,150,210,270,330].

### Patterns of persistence of bTB in Great Britain

**Figure 2:**
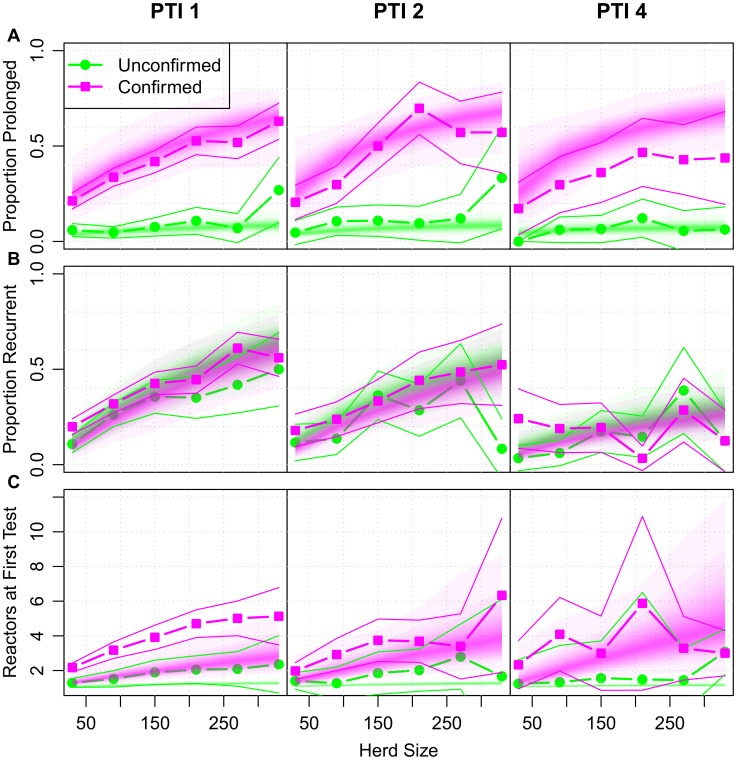
The persistence of bTB in GB herds (2003–2005). The within-herd persistence of bTB in GB as measured by the probability of all GB breakdowns from our study population being prolonged (duration of greater than 240 days, top panel) or recurrent within 24 months (middle panel). The relationship of each measure is plotted against herd size, with breakdowns further stratified by parish testing interval (PTI 1, 2 and 4, left to right) and confirmation status (unconfirmed breakdowns: lime green, circles, confirmed breakdowns: magenta squares). Uncertainty in each (mean) target observation (thick lines) is illustrated by an envelope (thin lines) of ±1.96 standard errors around the mean. Predictive distributions from our within-herd (SORI) model for each of these measures are plotted as shaded density strips where the intensity of color is proportional to the probability density at that point [35].

We further stratify these herds by the parish testing interval (PTI) and confirmation status of breakdowns to produce empirical distributions of persistence (Figure **2**, Figures **S6**,**S10**). This classification is motivated by the systematic differences in testing for herds within different PTIs and after confirmation. Confirmed (recently re-classified as Officially TB-Free Status Withdrawn or OTF-Withdrawn) breakdowns are required to pass an additional clear test at a more strict (severe) interpretation of the SICCT test (Figure **1B**). The severe interpretation increases the sensitivity of the SICCT test at the expense of reducing the specificity. Confirmation is triggered by the discovery of reactor animals with visible lesions and/or culture of *M. bovis*.

The proportion of prolonged and recurrent breakdowns both scale with herd size, but demonstrate distinct relationships with respect to both confirmation status and the local background risk of infection as measured by PTI. These empirical relationships are consistent with previous analyses suggesting that confirmation is associated with an increase in the duration of breakdowns [25], but has negligible impact on recurrence [10]. In contrast to the consistency of the duration of breakdowns across all areas there is a marked increase in the rate of recurrence with local risk as measured by PTI. These differential relationships of persistence with herd size are the basis on which we set out to infer the within-herd transmission dynamics of bTB.

### Modelling the persistence of bTB

We consider the persistence of bTB to be a product of the non-linear interaction of both the disease and testing dynamics. Heuristically, our model can therefore be considered as having two interacting dynamic components: an epidemic model that describes transmission within and into the herd and a testing model that models the sequence of tests and removal of reactors. We estimate the parameters of our model (Table **1**) directly from our target measures of persistence using a sequential Monte Carlo implementation of Approximate Bayesian Computation (ABC-SMC) [18], [19]. This framework is designed to bypass the calculation of computationally infeasible likelihood functions and instead generates distributions of parameters for which model outputs are consistent with the data according to a set of pre-defined goodness-of-fit metrics.

**Table 1:** Model parameters.

Parameter	Description
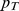	**Standard Sensitivity.** Probability of positive test from R,I compartments at standard definition.
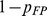	**Standard Specificity.** Probability of negative test from all compartments at standard definition.(1 – probability of a false positive 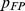 )
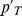	**Severe Sensitivity.** Probability of positive test from R,I compartments at severe definition
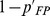	**Severe Specificity.** Probability of negative test from all compartments at severe definition.(1 – probability of false positive 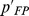 )
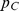	**Confirmation.** Probability of confirmation of breakdown per reactor based on slaughter-house inspection and culture (***only*** applies to reactors from O,R,I compartments)
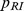	**Slaughterhouse routine inspection.** Probability of detecting an infected animal (O,R,I compartment) at slaughter under routine inspection
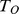	**Occult Period.** Mean length of time that animals are undetectable (occult) to SICCT
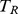	**Reactive Period.** Mean length of time between infection and animals becoming infectious
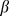	**Transmission parameter** associated with density dependence (rate per day, dimensions change with q)
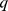	**Transmission parameter** measuring the strength of density dependence (range 0–1)
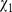	**Transmission parameter** measuring infectious pressure per susceptible per year in PTI 1
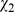	**Transmission parameter** measuring infectious pressure per susceptible per year in PTI 2
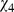	**Transmission parameter** measuring infectious pressure per susceptible per year in PTI 4
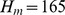	**Constant** equal to mid-point of range of herd sizes within study population. Used to transform density dependence of force of infection.

The period of latency between infection and infectiousness is a key epidemiological parameter that sets the time-scale between subsequent epidemic generations. Given the chronic, progressive nature of bTB, models have conventionally assumed long epidemiological latent periods of ∼6–20 months [13], [14]. However, animal challenge studies have suggested that bacterial shedding, and therefore transmission, may occur over shorter time-scales of ∼30 days [29]. In order to explore these two scenarios of latency, we fitted two models using vague (uniform) prior assumptions (Table **2**), one based on the conventional model structure assumed for bTB [13], [14] (SORI, Table **3**) and an alternative model allowing for “early” infectiousness (SOR, Table 4). In the SORI model cattle are assumed to be infectious (**I**) only after passing through two latent stages: an occult stage (**O**) where animals are infected, but not detectable by SICCT testing and a reactive stage (**R**) where animals are ‘reactive’ to the SICCT test but not yet infectious. The SOR model decouples this relationship between epidemiological latency and reactivity to the SICCT test. Animals are assumed to be potentially infectious from both the occult (**O**) and reactive (**R**) classes, dispensing with the infectious class (**I**).

**Table 2:** Prior assumptions.

Parameter	Prior Constraints	Initial sampling distribution
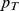	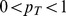	Uniform [0.05,1]
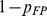	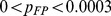	Uniform [0.05,1-0.9997]
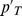	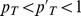	Uniform [0.4,1]
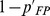	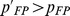	Uniform [0,1-0.9990]
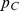	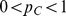	Uniform [0.0,0.5]
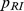	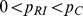	Uniform [0.0,0.5]
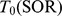	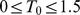	Uniform [0.0,1.5]
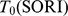	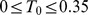	Uniform [0.0,0.35]
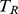	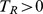	Uniform [0.0,1.5]
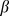	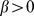	Uniform [0,2.0]
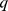	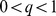	Uniform [0,1]
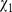	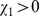	Uniform [0,3e-4]
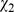	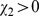	Uniform [0, 3e-4]
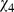	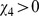	Uniform [0, 3e-5]

**Table 3:** Events defining SORI stochastic epidemic model.

Event	Effect	Probability per unit time
Move susceptible animal onto herd	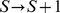	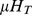
Remove animal from herd	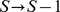	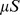
	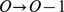	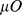
	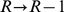	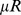
	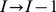	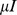
Transmission	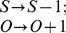	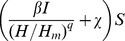
Emergence (Occult)	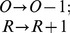	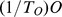
Emergence (Reactive)	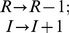	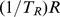

**Table 4:** Events defining SOR stochastic epidemic model.

Event	Effect	Probability per unit time
Move susceptible animal into herd	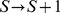	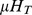
Remove animal from herd	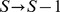	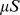
	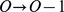	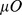
	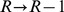	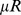
Transmission	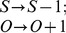	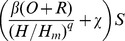
Emergence	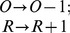	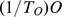

Both models provide comparable fits to the empirical target distributions (Figure **2**, Figures **S6**,**S10**) despite remarkably different estimates for the duration of the occult period and the epidemiological period of latency between infection and infectiousness (Figures **S5**, **S9**). The occult period is estimated to last ∼1.8 days (0.0–7.7 days, 95% CI) for the SOR model and ∼275 days (24–517, 95% CI) for the SORI model. Whereas infectiousness is implicitly assumed to begin with infection under the SOR model, the period of epidemiological latency estimated under the SORI model is more than twice that of previously assumed values [13], [14] with a point estimate of 406 days (116–827, 95% CI). The occult period estimated from the SORI model is an order of magnitude larger than the range of 8–65 days observed in animal challenge studies [12], [30], [31]. Placing a more informative prior (uniform on the range 0–128 days) on the occult period for the SORI model has no appreciable impact on the fidelity of the model fit and reduces the estimated occult period to a median point estimate of ∼28 days (1–119 days, 95% CI). We therefore select the SORI model fit with the more informative prior for comparison with the alternative SOR model within this paper.

**Figure 3:**
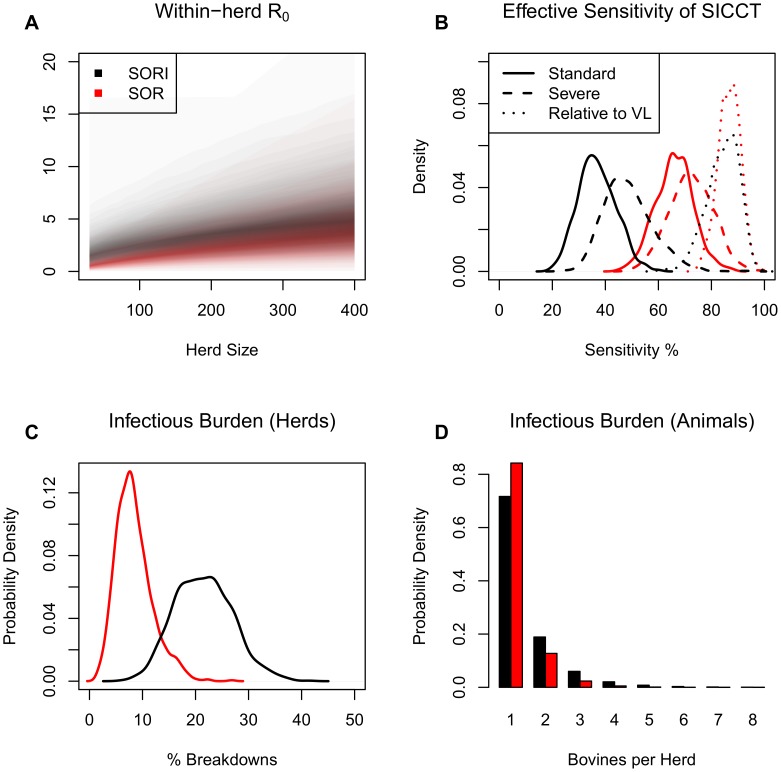
Herd level measures of efficiency of transmission and clearance of infection for SORI and SOR models. (**A**) Predictive distribution for the within-herd reproduction ratio (R_0_), plotted as a shaded density strip where the intensity of shading is proportional to the probability that R_0_ takes a given value [35]. We calculate 
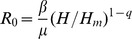
 where 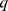 measures the strength of density dependence, 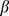 is the estimated transmission parameter and 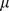 the per capita rate of turnover of the herd sampled from an empirical distribution and 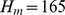. Both models estimate that transmission is non-linearly density dependent with point estimates for the invasion threshold (R_0_ = 1) of 71 (8–674,95% CI) for the SORI model and 12 (1–833, 95% CI) for the SOR model. (**B**) The effective sensitivity of the SICCT test within our study population of herds measured under the standard (solid line) and severe (dashed line) interpretations and relative to the gold standard of confirmation with visible lesions (dotted line). (**C,D**) The infectious burden remaining after movement restrictions are lifted can be characterized in terms of the number of herds with at least one infectious animal remaining when movement restrictions are lifted (**C**) and the expected distribution of animals on those herds (**D**). Predictive distributions for (**B,C,D**) are calculated by simulating from the empirical distribution of herds taken from our study population, representing the national distribution of herds.

Both models estimate that the rate of cattle-to-cattle transmission within a herd increases, non-linearly, with herd size. The potential for transmission within a herd can be characterized by the basic reproductive ratio R_0_, defined as the expected number of secondary cases within a herd of size N on the introduction of a single infectious individual. Within the range of our study population our (median) point estimate of R_0_ from the SORI model increases from 1.5 (0.26–4.9; 95% CI) in a herd of size 30 up to 4.9 (0.99–14.0; 95% CI) in a herd of 400 cattle (Figure **3A**). Estimates from the SOR model are smaller, but with overlapping credible intervals, increasing from 0.52 (0.1–1.6, 95% CI) in a herd of size 30 up to 3.6 (0.73–8.85, 95% CI) in a herd of size 400 (Figure **3A**). As a consequence, both models predict that the efficiency of control will also scale with herd size.

The SOR and SORI models provide contrasting estimates of the efficiency of SICCT testing in Great Britain. The SOR model estimates a true median SICCT test sensitivity of 66% (52–80%, 95% CI) at the standard interpretation, rising to 72% (56–88%, 95% CI) under the severe interpretation. Estimates of true sensitivity from the more traditional SORI model are far lower, at 36% (24–51%, 95% CI) for the standard interpretation rising to 48% (34–69%, 95% CI) for the severe interpretation (Figure **3B**). However, these model estimates are relative to the true infection status of animals. Given the lack of a gold standard diagnostic test for bTB, such information is not available in real populations. Rather, out of necessity, sensitivity of diagnostic tests for bTB is routinely measured relative to the presence of visible lesions and/or culture. The limitations of such comparative measures of sensitivity are aptly illustrated by comparison of our estimates from the SOR and SORI models. Despite the differences in the true sensitivity between the two models, the effective test sensitivities relative to visible lesions are indistinguishable and considerably higher than the true values (Figure **3B**). As a consequence the diverse sensitivity estimates from the SOR and SORI models are nonetheless both consistent with published estimates of the sensitivity of SICCT relative to visible lesions of up to 93.5% at the severe interpretation [11].

In order to quantify the efficiency of control we introduce a new measure - the infectious burden. We define infectious burden as the probability that *at least one* infected animal remains within a herd after movement restrictions are lifted. By simulating our within-herd models using the distribution of herd sizes from our study population we can generate predictive distributions for the infectious burden at the national level (Figure **3 C,D**). Once more, the SOR and SORI models provide contrasting views of the efficiency of control. Under the SOR model we estimate that 8% (3–17%; 95% CI) of breakdowns will have an infectious burden when they clear restrictions, with a median of 1 (1–3; 95% CI) infectious animal remaining in these herds. Under the SORI model this estimate increases to 21% (12–33%; 95% CI) of breakdowns with an infectious burden when they clear restrictions, with a median of 1 (1–4; 95% CI) infectious animal remaining in these herds.

### Implications for control

**Figure 4:**
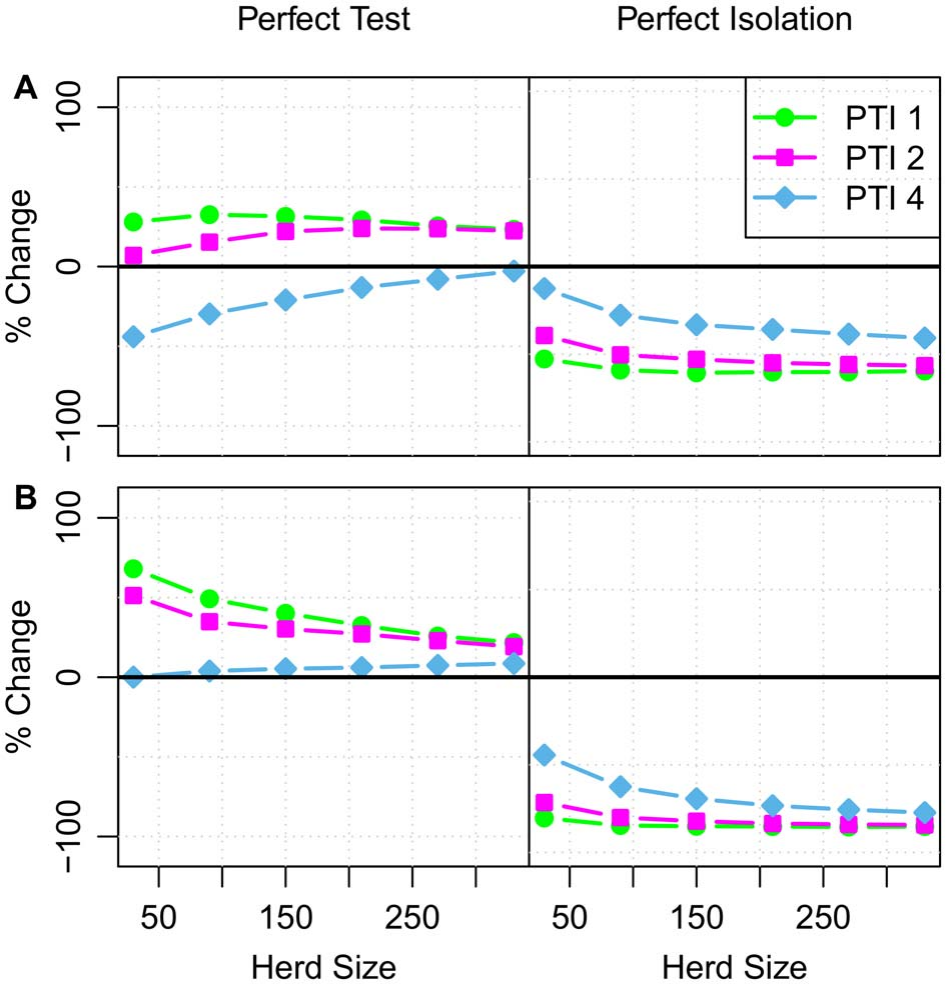
Impact of herd-level interventions on probability of recurrence within 24 months. Change in the probability of a herd experiencing a recurrent breakdown after application of a ‘perfect’ test (left column) or perfect isolation (right column). The perfect test is assumed to have 100% sensitivity and specificity and no occult period. Perfect isolation corresponds to setting the extrinsic infectious pressure to zero at the end of a breakdown (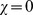). Plotted values correspond to the average % difference in the probability of recurrence relative to the fitted SORI (Panel **A**) and SOR models (Panel **B**). Separate series are plotted for herds in PTI 1 (lime green circles), 2 (magenta squares) & 4 (sky blue diamonds). Predictive distributions illustrating the variability in these point (mean) estimates are presented in supplementary figure **S13**.

We apply our fitted models to predict how different herd-level interventions may affect the resolution of breakdowns (Figure **4**). Specifically we consider two treatments: application of a ‘perfect test’ and eliminating the extrinsic infectious pressure through ‘perfect isolation’. In the SOR model, recurrence is driven almost completely by re-introduction, with ‘perfect isolation’ having the potential to eliminate recurrence completely in some herds (Figure **4B**). Perfect isolation is predicted to be less effective at reducing recurrence in herds with a low extrinsic rate of re-introduction (i.e. small herds in low incidence areas). Although these herds are predicted to have a lower infectious burden (Figures **S8**, **S12**), when they do experience a recurrent breakdown it is more likely to be caused by infection missed by SICCT testing than re-introduction.

Under the SORI model there is a similar relationship in the response to ‘perfect isolation’, except that a greater proportion of recurrence is attributable to persistence of infection. At the national level, averaging over our study population of herds once again, we estimate that 50% (33–67, 95% CI) of recurrent breakdowns are attributable to persistence within the SORI model, compared to 24% (11–42, 95% CI) under the SOR model.

However, eliminating this hidden burden of infection is not sufficient to eliminate recurrence if the extrinsic infectious pressure acting on herds is not simultaneously addressed. Under both models a ‘perfect test’ with 100% sensitivity, specificity and no occult period fails to improve the probability of recurrence in high incidence areas (Figure **4**). Although the perfect test reduces the duration of breakdowns, it can also detect infection within herds more quickly. In the short term, rates of recurrence will therefore increase in high incidence areas and such a perfect test would only be of benefit for low incidence (PTI 4) breakdowns where the rate of re-introduction is sufficiently low.

This counter-intuitive result demonstrates an important limitation of our approach. Our herd-level model does not distinguish between movements to slaughter or to other herds, so the infectious burden output from our model may potentially be contributing to the extrinsic rate of transmission that drives recurrence in our herd-level model. Both of our within-herd models can equally well fit the empirical patterns of persistence of bTB despite very different predictions for the level of the infectious burden. However, such a difference would place very different weights on the importance of cattle movements in network models of herd-to-herd transmission. Recent analyses of the between-herd transmission of the disease in GB have simplified, or ignored these within-herd dynamics of transmission [6], [7].

## Discussion

A fundamental challenge in epidemiological modeling concerns identifying the appropriate level of model complexity required to understand the dynamics of transmission and form a rational basis for policy development. Tuberculosis has been described as an infectious disease with a period of latency ranging from one day to a lifetime [32]. However, this uncertainty surrounding the progression of disease in individuals is rarely considered as a part of epidemiological modeling studies. In this study we have demonstrated how assumptions concerning the relative timing of infectiousness and reactivity to tuberculin profoundly impacts upon the estimated efficiency of SICCT testing.

Both the SOR and SORI models are equally well supported by the population level data used in this study, despite very different estimates for the efficiency of testing. This suggests that persistence measures alone are insufficient to distinguish the true burden of infection and points to experimental studies that could resolve this uncertainty. Neither model identifies, without the support of informative priors, an occult period within the range observed from animal challenge studies [12], [30], [31]. However, if there is a relationship between infectious dose and the duration of latency, estimates from challenge studies must also be treated with caution. Given the importance of this parameter in determining the hidden burden of infection, further research is required to clarify the relationship between infectiousness and sensitivity to diagnostic tests. Our modeling suggests that transmission of bTB ‘early’ in infection necessitates a lower level of persistence of infection than predicted by traditional (SORI) transmission models. However, evidence for such ‘early’ transmission comes from animal challenge studies [29], [33] and has not been verified under natural transmission conditions. Our modeling emphasizes the critical importance of understanding how the pattern of bacterial shedding in naturally infected animals changes over time.

Both models estimate that the rate of cattle-to-cattle transmission in GB herds is non-linearly density dependent. This result has immediate importance for the formulation of bTB policy at the herd level, suggesting that additional controls may need to be targeted towards larger herds. Our models suggest that the key to addressing the ongoing spread of bTB lies with reducing the rate of transmission into herds. The central question remains as to whether this requires management of the reservoir of infection in wildlife populations, or simply improved surveillance and diagnostic testing to reduce the movement of undisclosed infection between herds.

We have shown that stochastic persistence measures can provide insights into the efficiency of control measures for managed populations. However, the interpretation of these patterns of persistence requires a modeling approach that simultaneously accounts for the dynamics of control as well as the intrinsic dynamics of disease transmission. In the case of bTB, the dynamics of infection at the individual level have a profound impact on the estimated burden of infection missed by testing. It is therefore imperative to improve our understanding of the, still mysterious, life history of infection of bTB in individual cattle.

## Methods

### Epidemic model

Within-herd transmission of bTB is modeled using the standard compartmental approach where animals are classified only by their epidemiological status. We consider two alternative models (**SORI** and **SOR**) corresponding to different assumptions concerning the relationship between latency, transmission and reactivity to the SICCT skin test. In the traditional **SORI** model for bTB, occult (**O**) and reactive (**R**) animals are infected but not yet infectious, differing only in their response to the SICCT test. Infectious animals (**I**) are assumed to be both infectious and detectable by the SICCT test with the same efficiency as reactive animals. In the **SOR** model occult (O) and reactive (R) animals are both assumed to be potentially infectious eliminating the need for the infectious class (**I**).

Both epidemic models are implemented as stochastic Markov chains in continuous time and can be defined by the allowed transitions between the four state variables: Susceptible (**S**), Occult (**O**), Reactive (**R**) and Infectious (**I**) (Tables **3** **,** **4**). A per capita turnover rate μ is sampled from an empirical distribution for each simulation (Figure **S3**). A constant target herd size (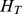) is maintained by balancing a constant per-capita removal rate (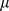) with a fixed import rate of 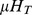. Herd size therefore fluctuates, with an instantaneous herd size of 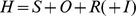. The extrinsic infectious pressure (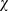) is the only parameter to vary with the parish testing interval (PTI) taking unique values for PTI 1, 2 and 4 (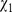,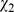,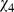).

### Testing model

The sequence of tests before, during and after a breakdown is simulated by a model where the timing of tests and number of animals to be tested changes dynamically according to the state-variables of the epidemic model and the outcome of individual animal tests.

Model simulations are initialised with the entire herd in the susceptible compartment (S,O,R,I) = (N, 0, 0, 0). The model is then simulated forward, piecewise, between the dynamically scheduled tests before, during and for 5 years following the end of the first breakdown, or until a recurrent breakdown is triggered. The sequence of decisions following the outcome of herd tests is summarized in Figure **1B**.

Simulations begin with the herd undergoing routine surveillance through slaughterhouse inspection and whole herd tests (classified as RHT or WHT) at 1, 2 or 4 yearly intervals (described below). Detection of a reactor animal triggers a breakdown. The herd then enters a sequence of short interval tests (SIT). Unconfirmed breakdowns end after a single clear test at the standard interpretation, while confirmed breakdowns must clear two tests – one at severe interpretation and the second at standard interpretation. Two follow-up tests, one six months after the end of a breakdown (VE-6M) and one 12 months later (VE-12M) are then scheduled. The time between all tests associated with a breakdown (SIT, VE-6M, VE-12M) are sampled from empirical distributions (Figure **S1**, Dataset **S1**). The duration of time between routine tests is also sampled from an empirical distribution (with separate distributions for PTI 1, 2 and 4) to account for the additional variation in the time to detection that is a consequence of delays in testing and the transition of herds between different parish testing intervals (Figure **S1**, Dataset **S1**).

Breakdowns are triggered by the detection of a reactor, either due to the presence of infected animals in the herd or the generation of a false positive test result. Nominally, we simulate the full sequence of tests until either of these events occurs with the proportion of false-positive breakdowns determined by the relative values of the specificity (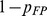) and the infectious pressure (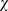). In practice, and to increase the speed of simulations, this can be pre-calculated by explicitly calculating the probability of a false positive breakdown occurring between periods where there are no infectious animals within the herd. Breakdowns can also be triggered by routine slaughterhouse surveillance that is modeled as a fixed probability (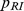) that removals from the **O**, **R** and **I** compartments will be detected. Breakdowns triggered by slaughterhouse surveillance are treated as confirmed breakdowns, with the first whole herd test carried out under the severe interpretation.

### Simulating herd tests

The application of herd tests in GB can be modeled by simulating three basic processes 1) the number of animals to test 2) the number of reactor animals detected at the standard and severe interpretations of the skin test and 3) the confirmation process.

For tests associated directly with a breakdown (SIT, VE-6M, VE-12M) the whole herd is tested. However, there is more variation in the type of test, and numbers of animals tested, in PTI 2 and 4 herds. We simulate this process by choosing the test type – either a whole herd test or a routine herd test – at random according to the proportion of tests recorded within the parish testing interval of the simulated herd (Figure **S2**, Dataset **S1**).

Whole herd tests specify that all bovines older than 6 weeks should be tested. We simulate this requirement by approximating the instantaneous proportion of the herd ineligible for testing to be (6/52) μ/N, where μ is the per-capita turnover of the herd. The number of animals tested with a WHT (X) is then sampled from a binomial distribution:
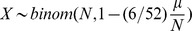



There is greater variability in which non-breeding animals are tested during a routine herd test (RHT), and therefore in the proportion of the herd tested. In order to account for this we sample the proportion from a Cauchy distribution fitted to the empirical distribution from VetNet data by maximum likelihood (Figure **S2**) with scale parameter 0.0932 and shift parameter 0.494 (to 3 s.f.). The number of animals tested with a RHT (X) is then sampled from a binomial distribution as before with:
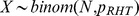


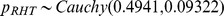



The outcome of diagnostic tests within our model is determined by the set of parameters defining the sensitivity and specificity of the SICCT test at both the standard and severe interpretations (Table **1**). In the field, the classification of reactors is based on cut-off values for the difference in reaction between avian and bovine tuberculin, with the cut-off value for a reactor changing with the severe and standard interpretation. As a consequence, tests can, and are, re-interpreted at the severe interpretation following the confirmation of a reactor animal after slaughter. In order to model the process of confirmation in a consistent fashion we must simulate the test outcome for each individual animal in the herd separately to ensure that the number of reactors at the severe interpretation is strictly greater than or equal to the number at the standard interpretation.

Given X animals to test we sample them randomly (and uniformly) from each of the model compartments (S,O,R,I) to generate the number of animals from each compartment that are tested (X_S_,X_O_,X_R_,X_I_). For each (X_S_,X_O_,X_R_,X_I_) we sample a uniform random number and use the value to simulate the number of reactor animals at the standard (Standard Reactors) and severe (Severe Reactors) interpretations:

For each X_S_:
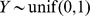



if (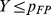): Standard Reactors +1;

if (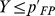) : Severe Reactors +1

For each X_o_:
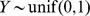



if (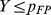) : Standard Reactors +1; Z+1

if (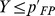) : Severe Reactors +1

For each X_R_:
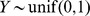



if (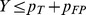) : Standard Reactors +1; Z+1

if (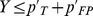) : Severe Reactors +1

For each X_I_:
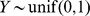



if (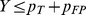) : Standard Reactors +1; Z+1

if (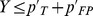) : Severe Reactors +1

We must also keep track of the number of true reactors (Z) in order to simulate the number of confirmed reactors (C):
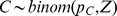



Provided that the breakdown has not been previously confirmed and C = 0, then all reactors at the standard interpretation are removed from the herd. Otherwise, if the number of confirmed reactors 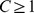, then the breakdown status is switched to confirmed (requiring an additional severe interpretation clear test to move back to the standard interpretation) and the reactors at the severe interpretation (including those from the current test) are removed.

### ABC-SMC implementation

We use the ABC-SMC algorithm described in Toni *et al.* [19]. In essence the method replaces the calculation of a likelihood function with an approximation based on matching model simulations to the observed data using a set of goodness-of-fit metrics (in this case corresponding to a set of key epidemiological target measures). These target measures can be combined to produce a single metric (described in the next section). For a given set of parameters, a series of stochastic simulations from the model are produced using the algorithm described in the previous section. A simulation is said to “match” the observed data if the corresponding (simulated) metric value lies below a pre-determined threshold. A Monte Carlo estimate of the probability of matching can then be used as an approximation to the likelihood in an SMC framework [18], [19]. As the tolerance applied to the metric is reduced, the approximate (posterior) distribution should in principle converge towards the (true) posterior distribution.

The algorithm begins by generating 10,000 *particles* - each “particle” corresponding to a set of model parameters – from a set of uniform proposal distributions (Table **2**). For each particle we produce a binary Monte Carlo estimate for the probability of matching. This information is combined with the prior distributions for the parameters to produce a set of weights across the whole population of particles. The algorithm then proceeds through a series of repeated steps, whereby the population of particles is re-sampled from the previous weighted population and then each particle perturbed according to a set perturbation kernel. A new set of weights is then generated in a similar manner to before. The tolerance controlling the matching is reduced at each step until the predictive distributions from the simulated model generate an acceptable agreement with the target epidemiological measures. All parameters are log transformed and the perturbation kernel is uniform for each parameter (on this scale) 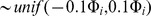, where 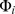 is the range of the marginal distribution for parameter 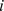 from the previous SMC round.

At each successive round the threshold was reduced semi-automatically to the median value of the metric from the previous round. Heuristically the ABC-SMC procedure can be thought of as using goodness-of-fit criteria to inform the shape of the approximate posterior distributions, rather than the likelihood function.

### Prior assumptions

Uniform prior distributions are applied to individual parameters and combinations of parameters to constrain their values to biologically relevant ranges. Probabilities are constrained to be in the interval [0,1] and rates are constrained to be positive. The sensitivity and specificity of the SICCT test are constrained to increase and decrease respectively under the severe interpretation and the probability of confirmation of reactors under routine slaughterhouse surveillance is assumed to be less than the probability of confirmation of reactors. All prior assumptions are intended to be uninformative, apart from the occult period for the SORI model and the upper bound (0.0003) placed on p_FP_. This value, equivalent to a lower bound on the specificity of the skin test at the standard definition of 99.97%, was obtained by calculating the value of p_FP_ required to explain *all* of the unconfirmed breakdowns within VetNet data given the total number of animal tests.

### Target epidemiological measures

Table **5** summarizes the target measures used to build our ABC metric. For each measure there are 6 empirical targets, by 2 values of confirmation status, by 3 values of PTI to give 36 target distributions/probabilities for each epidemiological measure.

**Table 5:** Epidemiological target measures for ABC.

Description	Type of Measure	Number of bins per target distribution	Weighting ( 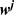 )
Breakdown Length	Distribution (Days) [100,200,300,400,500,1000,2000]	7	1/7
Reactors at first test	Distribution (Reactors) [1,2,3,4,5,10,47]	7	1/7
Reactors at VE-6M	Distribution (Reactors) [1,2,3,4,5,10,47]	7	1/7
Reactors at VE-12M	Distribution (Reactors) [1,2,3,4,5,10,47]	7	1/7
Total reactors removed within breakdown (until movement restrictions are lifted)	Distribution (Reactors) [2,4,6,8,10,12,14,16,18,20,47]	11	1/11
Probability of recurrence within 6 months	Probability	1	1
Probability of recurrence within 12 months	Probability	1	1
Probability of recurrence within 24 months	Probability	1	1

All of the target epidemiological measures for our final ABC-SMC scheme can be expressed either as probabilities or as (binned) probability distributions. This motivated the choice of an ABC metric based on the relative entropy, also known as the Kullback-Leibler divergence [34], that measures the distance between a proposed probability distribution (p) and a reference distribution (q):
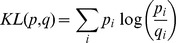



Two properties of the relative entropy should be noted: firstly, the relative entropy is asymmetric to the choice of reference distribution, with 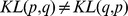. We choose to remove the potential ambiguity introduced through the choice of reference distribution by using a symmetrical metric (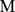):
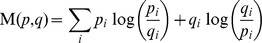



Secondly the relative entropy is undefined if any of the elements of the reference distribution q_i_ = 0. We numerically approximate the distribution of each of our target measures through histograms, with bin-sizes chosen to capture the range of observed values within VetNet data. Where we are free to choose appropriate bin sizes for the empirical distributions (q) such that we avoid any empty bins, we cannot ensure the same for the proposed distributions (p) generated from model simulations. To ensure that our metric is always defined, we add 1 to every bin of our empirical and simulated histograms.

For each proposed set of parameters (particle) we simulated a fixed number of realizations of the model (500) at the midpoint of each of the 6 herd-size histogram bins [30,90,150,210,270,330] for PTI 1,2 and 4 and generate a set of j proposed distributions (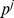) for each corresponding target measure (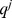). We use a superscript to indicate the jth distribution with elements indexed by i. We calculate the distance between the proposed and target distributions using the symmetric metric (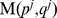) introduced above. The maximum value of this metric will increase with the number of histogram bins associated with that target distribution. In order to ensure that the overall metric 
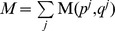
 places a more equal weight on each epidemiological measure we weight the contributions from each target measure 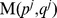 proportionally to the total number of bins forming that measure (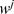) (Table **5**):
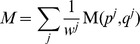



## Supporting Information

Dataset S1 Target and auxiliary distributions necessary for simulation and parameterization of models using ABC-SMC.
(ZIP)

Figure S1 Distribution of times between scheduled whole herd tests in GB (2003–2005). Farmers are responsible for scheduling tests as close as possible to the statutory intervals. Historically, this has lead to variation in the time between tests that we quantify for our study period (2003–2005). The frequency of routine herd tests is determined by the current parish-testing interval (PTI) for a herd. However, the time since the previous whole herd test is also determined by the historical testing intervals for the parish and other epidemiological factors. In practice the time since the previous surveillance test for breakdown herds in PTI 1, 2 and 4 is distributed with the greatest variation seen in PTI 2 (left). Short interval tests (SIT) must be carried out at least 60 days after the last whole herd test leading to a skewed distribution where test intervals are more likely to be late than early (middle). Likewise the follow up tests after a breakdown, that must be scheduled at intervals of at least 6 and 12 months respectively (VE-6 M, VE-12 M) are skewed to be late (right).
(PDF)

Figure S2 Proportion of animals tested in routine surveillance tests (2003–2005). The number of animals within a herd that are tested during routine surveillance varies depending on the demographic structure of the herd and the perceived epidemiological risk. PTI 1 herds should receive a whole herd test (WHT) where all bovines older than 6 weeks are tested. In PTI 2, 3 and 4 a routine herd test (RHT) may be carried out where there is greater discretion as to which animals are tested based on perceived epidemiological risk. As a consequence the proportion of animals tested is smaller and more variable for RHTs (Right) as compared to WHTs (Middle). The proportion of breakdowns reported as being disclosed by WHTs and RHTs also varies by PTI (Left table). The small proportion of breakdowns in PTI 1 initiated by a RHT, despite WHTs being mandated in these herds, stem from herds whose PTI was updated retrospectively after disclosure. Likewise despite the majority of tests in PTI 4 being RHTs, a higher proportion of *breakdown herds* are initiated by WHTs, applied when there was a perceived higher risk or consequence of infection (e.g. in dealer or milk retailer herds).
(PDF)

Figure S3 Annual per bovine rate of turnover in breakdown herds. We define turnover as the time-averaged rate at which animals move into and are removed from a herd. A representative distribution of turnover rates for breakdown herds was calculated from CTS data from 1^st^ January 2003 – 1^st^ January 2005 for all breakdown herds with start dates in 2004. Note that since the CTS data only records movements at the holding (CPH) rather than the herd (CPHH) level, this is an indirect measure corresponding to the annual per capita rate of movement of bovines through the CPH associated with a breakdown herd. Turnover was calculated three ways: using movements into a CPH (“On Movements”, red line), movements out of a CPH (“Off Movements”, green line) and the combined rate of both types of movements (black line, points). The median number of “Off” movements is slightly smaller with than for on-movements consistent with the increase in herd size nationally over the period. The “All movements” estimate is used as the empirical distribution for the within-herd model.
(PDF)

Figure S4 Distribution of Breakdown Herd Sizes. Smoothed density of the (maximum) herd size during a breakdown for our study population. There is some variation in the size of herds with PTI, with a longer tail of herds beyond our cutoff value of 360 (vertical dashed line) for PTI 4 herds.
(PDF)

Figure S5 Estimated Parameter Distributions for SORI model. Distributions of parameters consistent with the persistence measures and reactor distributions estimated from VetNet data (Figure **S6**). The severe values of sensitivity and specificity are constrained to be greater than and less than their respective values at the standard interpretation. Likewise the probability of infected animals being detected by routine slaughterhouse surveillance is assumed to be less than or equal to the probability of confirmation. All parameters are constrained to be positive, with probabilities and the density dependent parameter q further constrained to be less than or equal to 1.
(PDF)

Figure S6 Target measures for the within-herd persistence of bTB (2003–2005) and predictive distributions for SORI model. The within-herd persistence of bTB in GB as measured by the probability of breakdowns being prolonged (duration of greater than 240 days) or recurrent within a 6, 12 and 24 month horizon. The relationship of each target measure is plotted against herd size, with breakdowns further stratified by parish testing interval (PTI 1 top row, PTI 2 middle row, PTI 4 bottom row) and confirmation status (confirmed breakdowns: green, circles, unconfirmed breakdowns: magenta squares). Target measures are calculated from breakdowns trigged within 2003–2005 by a routine surveillance test (VE-WHT, VE-WHT2, VE-RHT, VE-SLH). The probability of confirmation varies between PTI, as does the proportion of confirmed breakdowns initiated by a slaughterhouse case (white diamonds) and the mean number of reactors reported at the disclosing test. Uncertainty in each (mean) target observation (thick lines) is illustrated by an envelope (thin lines) of ±1.96 standard errors around the mean. Predictive distributions for each of these target measures from the finalized within-herd transmission model are plotted as shaded density strips where the intensity of color is proportional to the probability density at that point.
(PDF)

Figure S7 Target measures for the within-herd persistence of bTB (2006–2008) and predictive distributions for SORI model. The within-herd persistence of bTB in GB as measured by the probability of breakdowns being prolonged (duration of greater than 240 days) or recurrent within a 6, 12 and 24 month horizon. The relationship of each target measure is plotted against herd size, with breakdowns further stratified by parish testing interval (PTI 1 top row, PTI 2 middle row, PTI 4 bottom row) and confirmation status (confirmed breakdowns: green, circles, unconfirmed breakdowns: magenta squares). Target measures are calculated from breakdowns trigged within 2003–2005 by a routine surveillance test (VE-WHT, VE-WHT2, VE-RHT, VE-SLH). The probability of confirmation varies between PTI, as does the proportion of confirmed breakdowns initiated by a slaughterhouse case (white diamonds) and the mean number of reactors reported at the disclosing test. Uncertainty in each (mean) target observation (thick lines) is illustrated by an envelope (thin lines) of ±1.96 standard errors around the mean. Predictive distributions for each of these target measures from the finalized within-herd transmission model are plotted as shaded density strips where the intensity of color is proportional to the probability density at that point.
(PDF)

Figure S8 Burden remaining after resolution of a breakdown using SORI model. redictive distributions for the probability of at least one infectious bovine remaining within a herd after a breakdown is resolved as a function of herd size, classified by PTI (1,2,4 left to right) and confirmation status (Green circles confirmed, magenta squares unconfirmed). Predictive distributions are plotted as shaded density strips where the intensity of shading is proportional to the probability density at that point. Solid lines, and points for each herd-size category, indicate the median of the predictive distribution to aid comparison between confirmed and unconfirmed breakdowns.
(PDF)

Figure S9 Estimated Parameter Distributions from SOR model. Distributions of parameters consistent with the persistence measures and reactor distributions estimated from VetNet data (Figure **S7**). The severe values of sensitivity and specificity are constrained to be greater than and less than their respective values at the standard interpretation. Likewise the probability of infected animals being detected by routine slaughterhouse surveillance is assumed to be less than or equal to the probability of confirmation. All parameters are constrained to be positive, with probabilities and the density dependent parameter q further constrained to be less than or equal to 1.
(PDF)

Figure S10 Target measures for the within-herd persistence of bTB (2003–2005) and predictive distributions for within-herd transmission model using SOR model. The within-herd persistence of bTB in GB as measured by the probability of breakdowns being prolonged (duration of greater than 240 days) or recurrent within a 6, 12 and 24 month horizon. The relationship of each target measure is plotted against herd size, with breakdowns further stratified by parish testing interval (PTI 1 top row, PTI 2 middle row, PTI 4 bottom row) and confirmation status (confirmed breakdowns: green, circles, unconfirmed breakdowns: magenta squares). Target measures are calculated from breakdowns trigged within 2003–2005 by a routine surveillance test (VE-WHT, VE-WHT2, VE-RHT, VE-SLH). The probability of confirmation varies between PTI, as does the proportion of confirmed breakdowns initiated by a slaughterhouse case (white diamonds) and the mean number of reactors reported at the disclosing test. Uncertainty in each (mean) target observation (thick lines) is illustrated by an envelope (thin lines) of ±1.96 standard errors around the mean. Predictive distributions for each of these target measures from the finalized within-herd transmission model are plotted as shaded density strips where the intensity of color is proportional to the probability density at that point.
(PDF)

Figure S11 Target measures for the within-herd persistence of bTB (2006–2008) and predictive distributions for within-herd transmission model using SOR model. The within-herd persistence of bTB in GB as measured by the probability of breakdowns being prolonged (duration of greater than 240 days) or recurrent within a 6, 12 and 24 month horizon. The relationship of each target measure is plotted against herd size, with breakdowns further stratified by parish testing interval (PTI 1 top row, PTI 2 middle row, PTI 4 bottom row) and confirmation status (confirmed breakdowns: green, circles, unconfirmed breakdowns: magenta squares). Target measures are calculated from breakdowns trigged within 2003–2005 by a routine surveillance test (VE-WHT, VE-WHT2, VE-RHT, VE-SLH). The probability of confirmation varies between PTI, as does the proportion of confirmed breakdowns initiated by a slaughterhouse case (white diamonds) and the mean number of reactors reported at the disclosing test. Uncertainty in each (mean) target observation (thick lines) is illustrated by an envelope (thin lines) of ±1.96 standard errors around the mean. Predictive distributions for each of these target measures from the finalized within-herd transmission model are plotted as shaded density strips where the intensity of color is proportional to the probability density at that point.
(PDF)

Figure S12 Burden remaining after resolution of a breakdown using SOR model. Predictive distributions for the probability of at least one infectious bovine remaining within a herd after a breakdown is resolved as a function of herd size, classified by PTI (1,2,4 left to right) and confirmation status (Green circles confirmed, magenta squares unconfirmed). Predictive distributions are plotted as shaded density strips where the intensity of shading is proportional to the probability density at that point. Solid lines, and points for each herd-size category indicate the median of predictive distribution to aid comparison between confirmed and unconfirmed breakdowns.
(PDF)

Figure S13Impact of herd-level interventions on probability of recurrence within 24 months. Change in the probability of a herd experiencing a recurrent breakdown after application of a ‘perfect’ test (left column) or perfect isolation (right column). The perfect test is assumed to have 100% sensitivity and specificity and no occult period. Perfect isolation corresponds to setting the extrinsic infectious pressure to zero at the end of a breakdown (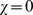). Plotted values correspond to the average % difference in the probability of recurrence relative to the fitted SORI (Panel **A**) and SOR models (Panel **B**). Separate series are plotted for herds in PTI 1 (lime green circles), 2 (magenta squares) & 4 (sky blue diamonds). Predictive distributions are plotted as shaded density strips where the intensity of shading is proportional to the probability density at that point, with the mean of the predictive distribution plotted as a solid line.(PDF)
