# Supplementary material for: Estimating the Hidden Burden of Bovine Tuberculosis in Great Britain
Source: PLoS Comput Biol. 2012 Oct 18;8(10):e1002730. doi: 10.1371/journal.pcbi.1002730 (PMC3475695; doi:10.1371/journal.pcbi.1002730)
Supplement: Dataset S1 — Target and auxiliary distributions necessary for simulation and parameterization of models using ABC-SMC. (ZIP) [file pcbi.1002730.s001.zip › Data Description.pdf]

Data Supplement

The formulation of our within-herd model depends on empirical distributions that describe the demographic turnover of herds and the interval between various surveillance tests. In the interests of reproducibility, we include these distributions as data-supplements to this paper along with measures of persistence used as targets for ABC. For reasons of data-confidentiality we only provide these processed outputs rather than the original records. In this document we describe the format of these data files **(Sections 2,3)** and sketch the prescription used to compile them from the raw data held by the Animal Health Veterinary Laboratory Agency.

Compiling these distributions required linking between surveillance records held within the VetNet database and demographic information held within the cattle tracing system (CTS) database. To understand the extent, and limitations, of these data we briefly describe the fields extracted from these databases and used in subsequent data analyses **(Section 1)**.

1) Overview of VetNet Surveillance data

Two key tables were extracted form the VetNet database that we will refer to as the *Breakdown* and *Testing Tables*.

Breakdown Table

The Breakdown table contains aggregate information for each bTB breakdown indexed by a unique identification number (BreakID). Additional information on the demography of breakdown herds (MaxHerdSize, HerdSize Start) were integrated into the breakdown table by cross-referencing herds with the CTS database. It should be noted that the CTS data contains records only up to the level of the county/parish/holding level (CPH), while the breakdown and testing tables record the full county/parish/holding/herd (CPHH). Due to variations in recording practices all estimates of herd size, including records from the CTS database involve some element of approximation. For consistency we use the maximum herd size during a breakdown (derived from CTS records) as our measure of herd size.

Fields available for each breakdown used for our analyses:

BreakID: Unique identification number for each breakdown

Cphh:	Unique reference number for each herd in GB (County/Parish/holding/herd)
BreakDate:	Date of start of Breakdown (i.e. Imposition of movement restrictions)
ConfFin:	Boolean variable, true if breakdown was confirmed at any point while under movement restrictions
MaxHerdSize:	Maximum herd size during the period of movement restrictions
NumCattleTested:	Number of animals tested during breakdown
NumCattleReactors:	Number of reactor animals removed from herd during movement restrictions
FirstTest:	Classification of Test that triggered breakdown
PTI:	Parish testing interval at start of breakdown
HerdSize_Start:	Herd Size on start date of breakdown
Reactors_Start:	Number of Reactors at disclosing test

Testing Table

The testing table records the results of each visit to a premise for the purpose of animal testing. Records correspond to a mix of whole herd and individual animal test records. A further complication is that larger herds may have multiple records corresponding to "part" tests carried out on successive days. Linking individual test records to breakdowns therefore often requires aggregation over multiple test records.

Fields from the testing table used for our analyses:

Cphh:	Unique reference number for each herd in GB (County/Parish/holding/herd)
TestDate:	Date of test
TestType:	Classification of test
Number:	Number of animals tested
Size:	Number of animals in herd
Reactors:	Number of reactor animals removed from herd
BreakID:	Unique identification number of breakdown (if test is associated with a breakdown)
Confirmed:	No. of Confirmed Reactors removed by testing

2) Testing Distributions

Routine Surveillance Tests (WHT/RHT)

Filename	Dimensions	(Type, units)
PTI1_Times.csv	[1681]	(integer, days)
PTI2_Times.csv	[458]	(integer, days)
PTI4_Times.csv	[285]	(integer, days)

Description

For each breakdown with start date within 2003-2005 (inclusive) the disclosing test was located within the Testing Table and the previous test record for that herd inspected. Providing the previous test was a routine surveillance test or a clear test terminating a previous breakdown (VE-WHT, VE-WHT2, VE-RHT, VE-12M, VE-CON) the difference between the start date (BreakDate) and the date (TestDate) of the previous test was recorded and binned by parish testing interval (PTI).

Short Interval Tests (SIT)

Filename	Dimensions	(Type, units)
SIT.csv	[882]	(integer, days)

Description

For each breakdown with start date within 2003-2005 (inclusive) and disclosing test classification of (VE-WHT,VE-RHT), the duration of time between successive short interval tests until resolution of the breakdown were recorded.

VE-6M Tests

Filename	Dimensions	(Type, units)
VE-6M.csv	[8048]	(integer, days)

Description

For each VE-6M test carried out within 2003-2005 (inclusive) the difference between the date of the VE-6M test (TestDate) and previous short interval test (VE-SIT) was calculated and recorded.

VE-12M Tests

Filename	Dimensions	(Type, units)
VE-12M.csv	[4261]	(integer, days)

Description

For each VE-12M test carried out within 2003-2005 (inclusive) the difference between the date of the VE-12M test (TestDate) and previous (VE-6M) test was calculated and recorded.

Turnover rates for herds

Filename	Dimensions	(Type, units)
Turnover.csv	[3286]	(floating point, per capita per year)

Description

The per-capita turnover was calculated for herds with breakdowns with start dates between 1/01/2004 and 1/01/2005 using the time-series of off and on movements between 1/01/2003-1/01/2006. Turnover was estimated as the gradient of a regression of cumulative exports (off movements from the CPH containing the herd) against time and is expressed as a per capita rate per year.

3) Target Distributions

Persistence Measures

Filename	Dimensions	(Type, units)
PersistenceTargets.csv	[37,9]	(floating point)

Fields

Herd Size:	Midpoint of histogram bin for Herd Size (Maximum over Breakdown)
PTI:	Parish Testing Interval
Confirmed:	Boolean variable with 1 indicating record for confirmed breakdowns.
Breakdowns:	Total number of binned breakdowns for specified herd size, PTI and confirmation status
Prolonged:	Number of breakdowns in bin with breakdown length greater than 240 days
Recur 6M:	Number of breakdowns in bin that experienced a recurrent breakdown within 6 months
Recur 12M:	Number of breakdowns in bin that experienced a recurrent breakdown within 12 months
Recur 24M:	Number of breakdowns in bin that experienced a recurrent breakdown within 24 months
Slaughterhouse:	Number of breakdowns in bin that were initiated by detection of a carcass with visible lesions at slaughter

Reactor Distributions

Filename	Dimensions	(Type, units)
ReactorsAtFirstTest.csv	[37,51]	(floating point)

Fields

Herd Size:	Midpoint of histogram bin for Herd Size (Maximum over Breakdown)
PTI:	Parish Testing Interval
Confirmed:	Boolean variable with 1 indicating record for confirmed breakdowns.
Breakdowns:	Total number of binned breakdowns for specified herd size, PTI and confirmation status

1-47: Number of breakdowns with given number of reactors at disclosing test

Filename	Dimensions	(Type, units)
TotalReactors.csv	[37,51]	(floating point)

Fields

Herd Size:	Midpoint of histogram bin for Herd Size (Maximum over Breakdown)
PTI:	Parish Testing Interval
Confirmed:	Boolean variable with 1 indicating record for confirmed breakdowns.
Breakdowns:	Total number of binned breakdowns for specified herd size, PTI and confirmation status
1-47:	Number of breakdowns with given number of reactors removed during movement restrictions (breakdown)

Filename	Dimensions	(Type, units)
ReactorsAtVE6M.csv	[37,51]	(floating point)

Fields

Herd Size:	Midpoint of histogram bin for Herd Size (Maximum over Breakdown)
PTI:	Parish Testing Interval
Confirmed:	Boolean variable with 1 indicating record for confirmed breakdowns.
Breakdowns:	Total number of binned breakdowns for specified herd size, PTI and confirmation status that failed a VE-6M test.
1-47:	Number of breakdowns with given number of reactors at VE-6M test.

Filename	Dimensions	(Type, units)
ReactorsAtVE12M.csv	[37,51]	(floating point)

Fields

Herd Size:	Midpoint of histogram bin for Herd Size (Maximum over Breakdown)

PTI:	Parish Testing Interval
Confirmed:	Boolean variable with 1 indicating record for confirmed breakdowns.
Breakdowns:	Total number of binned breakdowns for specified herd size, PTI and confirmation status that failed a VE-12M test.
1-47:	Number of breakdowns with given number of reactors at VE-12M test.

Filename	Dimensions	(Type, units)
ReactorsAtVE24M.csv	[37,51]	(floating point)

Fields

Herd Size:	Midpoint of histogram bin for Herd Size (Maximum over Breakdown)
PTI:	Parish Testing Interval
Confirmed:	Boolean variable with 1 indicating record for confirmed breakdowns.
Breakdowns:	Total number of binned breakdowns for specified herd size, PTI and confirmation status that failed a VE-24M test.
1-47:	Number of breakdowns with given number of reactors at VE-24M test.

Breakdown Length

Filename	Dimensions	(Type, units)
BreakdownLength.csv	[37,1902]	(floating point)

Fields

Herd Size:	Midpoint of histogram bin for Herd Size (Maximum over Breakdown)
PTI:	Parish Testing Interval
Confirmed:	Boolean variable with 1 indicating record for confirmed breakdowns.
Breakdowns:	Total number of binned breakdowns for specified herd size, PTI and confirmation status

1-1898:	Number of breakdowns with given duration of breakdown in days
