# Supplementary figures and images for: Estimating the Hidden Burden of Bovine Tuberculosis in Great Britain
Source: PLoS Comput Biol. 2012 Oct 18;8(10):e1002730. doi: 10.1371/journal.pcbi.1002730 (PMC3475695; doi:10.1371/journal.pcbi.1002730)

Time since last routine herd test

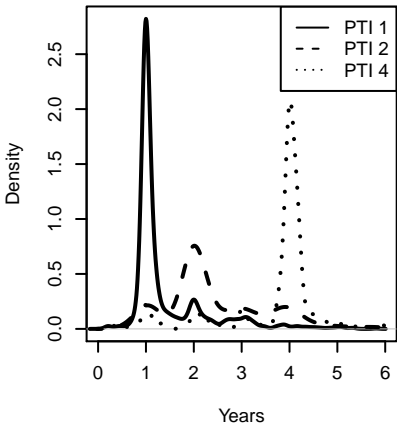

Time between SIT tests

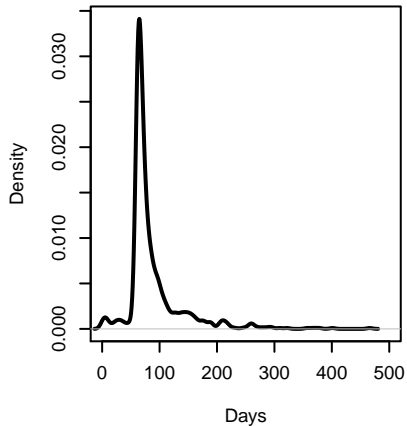

Time to follow-up tests

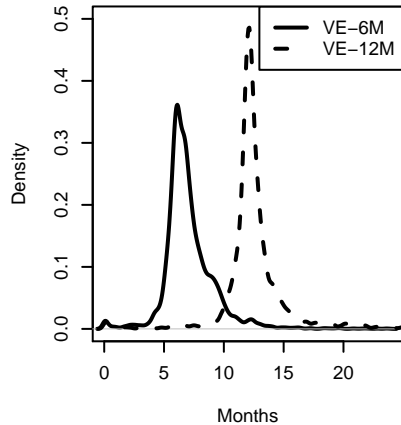

Supplement: Figure S1 — Distribution of times between scheduled whole herd tests in GB (2003–2005). Farmers are responsible for scheduling tests as close as possible to the statutory intervals. Historically, this has lead to variation in the time between tests that we quantify for our study period (2003–2005). The frequency of routine herd tests is determined by the current parish-testing interval (PTI) for a herd. However, the time since the previous whole herd test is also determined by the historical testing intervals for the parish and other epidemiological factors. In practice the time since the previous surveillance test for breakdown herds in PTI 1, 2 and 4 is distributed with the greatest variation seen in PTI 2 (left). Short interval tests (SIT) must be carried out at least 60 days after the last whole herd test leading to a skewed distribution where test intervals are more likely to be late than early (middle). Likewise the follow up tests after a breakdown, that must be scheduled at intervals of at least 6 and 12 months respectively (VE-6 M, VE-12 M) are skewed to be late (right). (PDF) [file pcbi.1002730.s002.pdf]

|       | WHT<br>(% Herds) | RHT<br>(% Herds) |
|-------|------------------|------------------|
| PTI 1 | 98%              | 1.9%             |
| PTI 2 | 50%              | 50%              |
| PTI 4 | 9.2%             | 91%              |

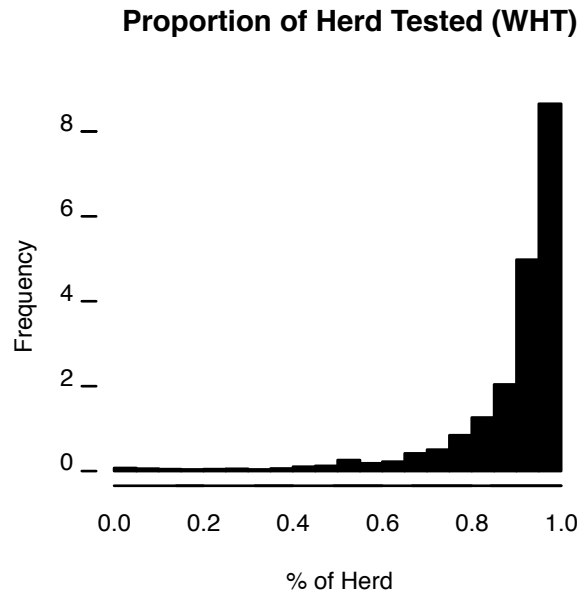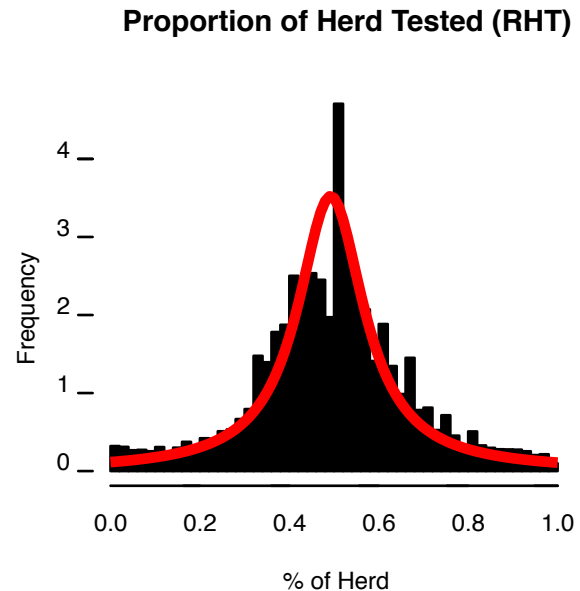

Supplement: Figure S2 — Proportion of animals tested in routine surveillance tests (2003–2005). The number of animals within a herd that are tested during routine surveillance varies depending on the demographic structure of the herd and the perceived epidemiological risk. PTI 1 herds should receive a whole herd test (WHT) where all bovines older than 6 weeks are tested. In PTI 2, 3 and 4 a routine herd test (RHT) may be carried out where there is greater discretion as to which animals are tested based on perceived epidemiological risk. As a consequence the proportion of animals tested is smaller and more variable for RHTs (Right) as compared to WHTs (Middle). The proportion of breakdowns reported as being disclosed by WHTs and RHTs also varies by PTI (Left table). The small proportion of breakdowns in PTI 1 initiated by a RHT, despite WHTs being mandated in these herds, stem from herds whose PTI was updated retrospectively after disclosure. Likewise despite the majority of tests in PTI 4 being RHTs, a higher proportion of breakdown herds are initiated by WHTs, applied when there was a perceived higher risk or consequence of infection (e.g. in dealer or milk retailer herds). (PDF) [file pcbi.1002730.s003.pdf]

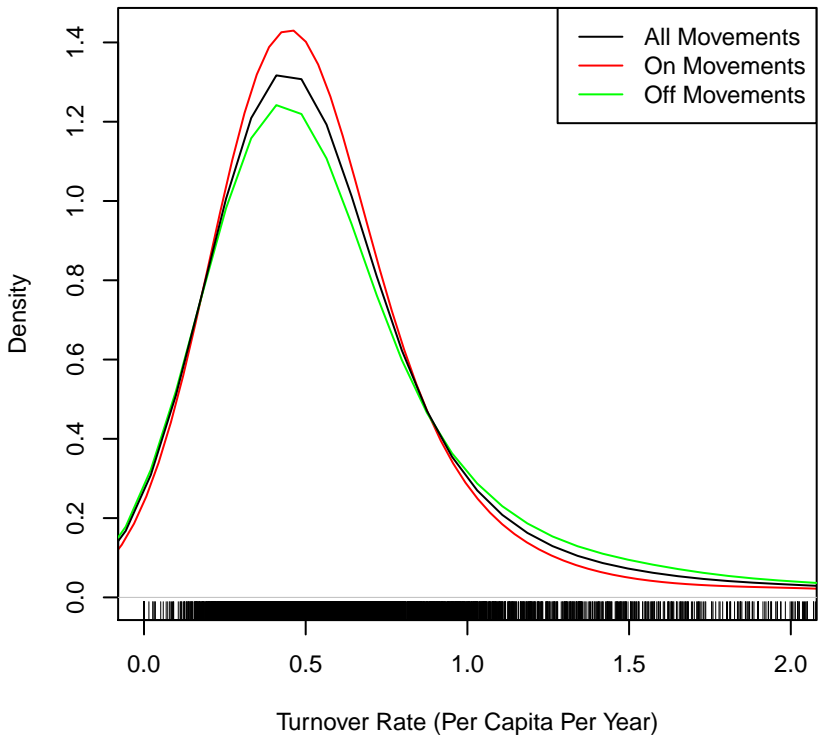

Supplement: Figure S3 — Annual per bovine rate of turnover in breakdown herds. We define turnover as the time-averaged rate at which animals move into and are removed from a herd. A representative distribution of turnover rates for breakdown herds was calculated from CTS data from 1st January 2003 – 1st January 2005 for all breakdown herds with start dates in 2004. Note that since the CTS data only records movements at the holding (CPH) rather than the herd (CPHH) level, this is an indirect measure corresponding to the annual per capita rate of movement of bovines through the CPH associated with a breakdown herd. Turnover was calculated three ways: using movements into a CPH (“On Movements”, red line), movements out of a CPH (“Off Movements”, green line) and the combined rate of both types of movements (black line, points). The median number of “Off” movements is slightly smaller with than for on-movements consistent with the increase in herd size nationally over the period. The “All movements” estimate is used as the empirical distribution for the within-herd model. (PDF) [file pcbi.1002730.s004.pdf]

## Size of Breakdown Herds (2002–2005)

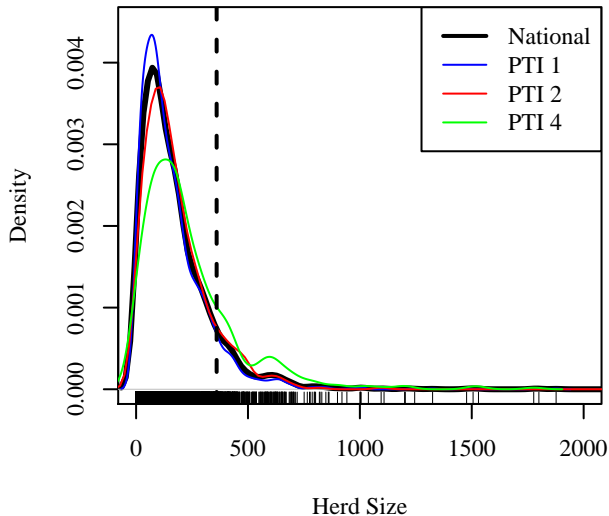

Supplement: Figure S4 — Distribution of Breakdown Herd Sizes. Smoothed density of the (maximum) herd size during a breakdown for our study population. There is some variation in the size of herds with PTI, with a longer tail of herds beyond our cutoff value of 360 (vertical dashed line) for PTI 4 herds. (PDF) [file pcbi.1002730.s005.pdf]

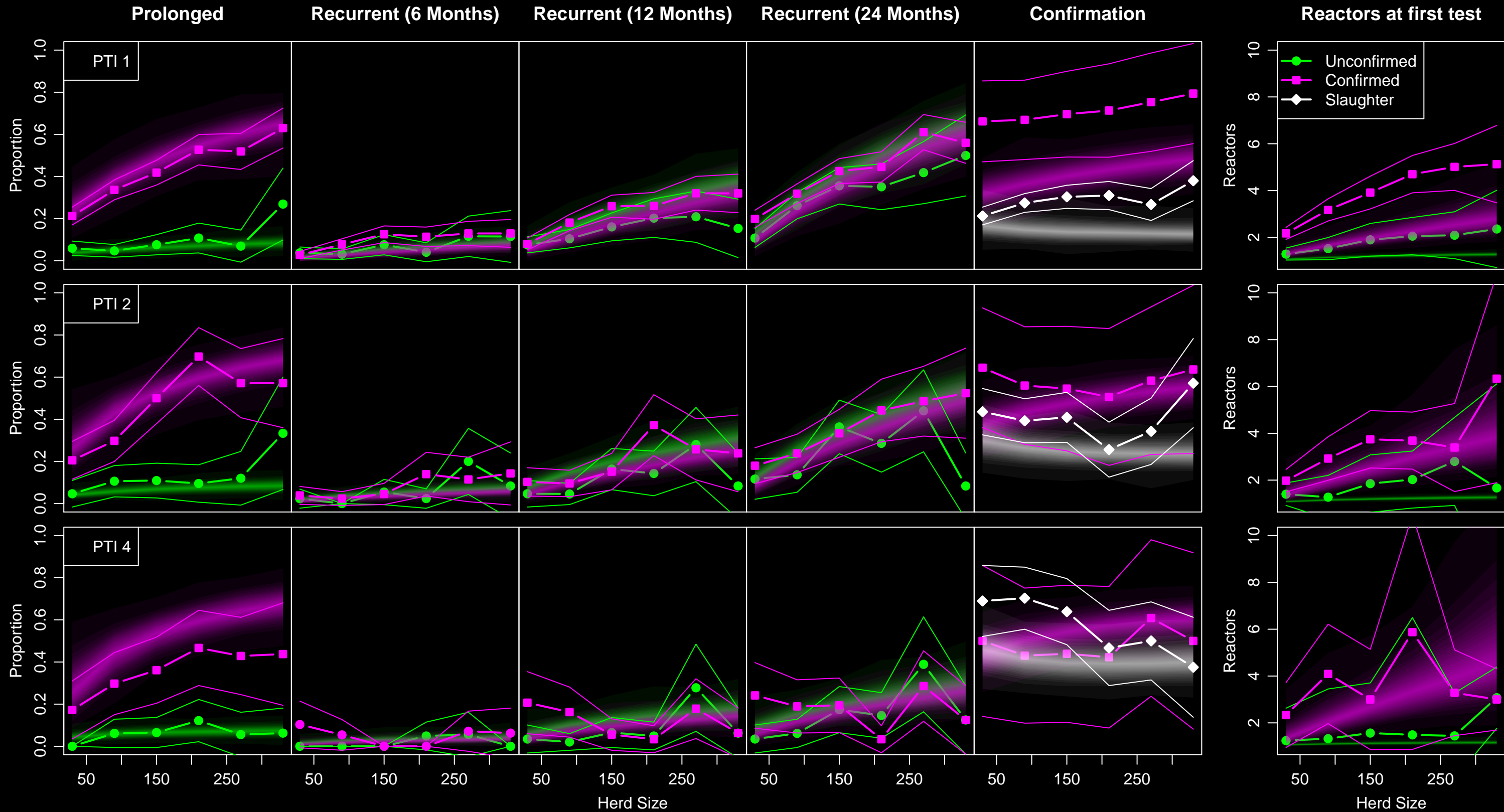

Supplement: Figure S6 — Target measures for the within-herd persistence of bTB (2003–2005) and predictive distributions for SORI model. The within-herd persistence of bTB in GB as measured by the probability of breakdowns being prolonged (duration of greater than 240 days) or recurrent within a 6, 12 and 24 month horizon. The relationship of each target measure is plotted against herd size, with breakdowns further stratified by parish testing interval (PTI 1 top row, PTI 2 middle row, PTI 4 bottom row) and confirmation status (confirmed breakdowns: green, circles, unconfirmed breakdowns: magenta squares). Target measures are calculated from breakdowns trigged within 2003–2005 by a routine surveillance test (VE-WHT, VE-WHT2, VE-RHT, VE-SLH). The probability of confirmation varies between PTI, as does the proportion of confirmed breakdowns initiated by a slaughterhouse case (white diamonds) and the mean number of reactors reported at the disclosing test. Uncertainty in each (mean) target observation (thick lines) is illustrated by an envelope (thin lines) of ±1.96 standard errors around the mean. Predictive distributions for each of these target measures from the finalized within-herd transmission model are plotted as shaded density strips where the intensity of color is proportional to the probability density at that point. (PDF) [file pcbi.1002730.s007.pdf]

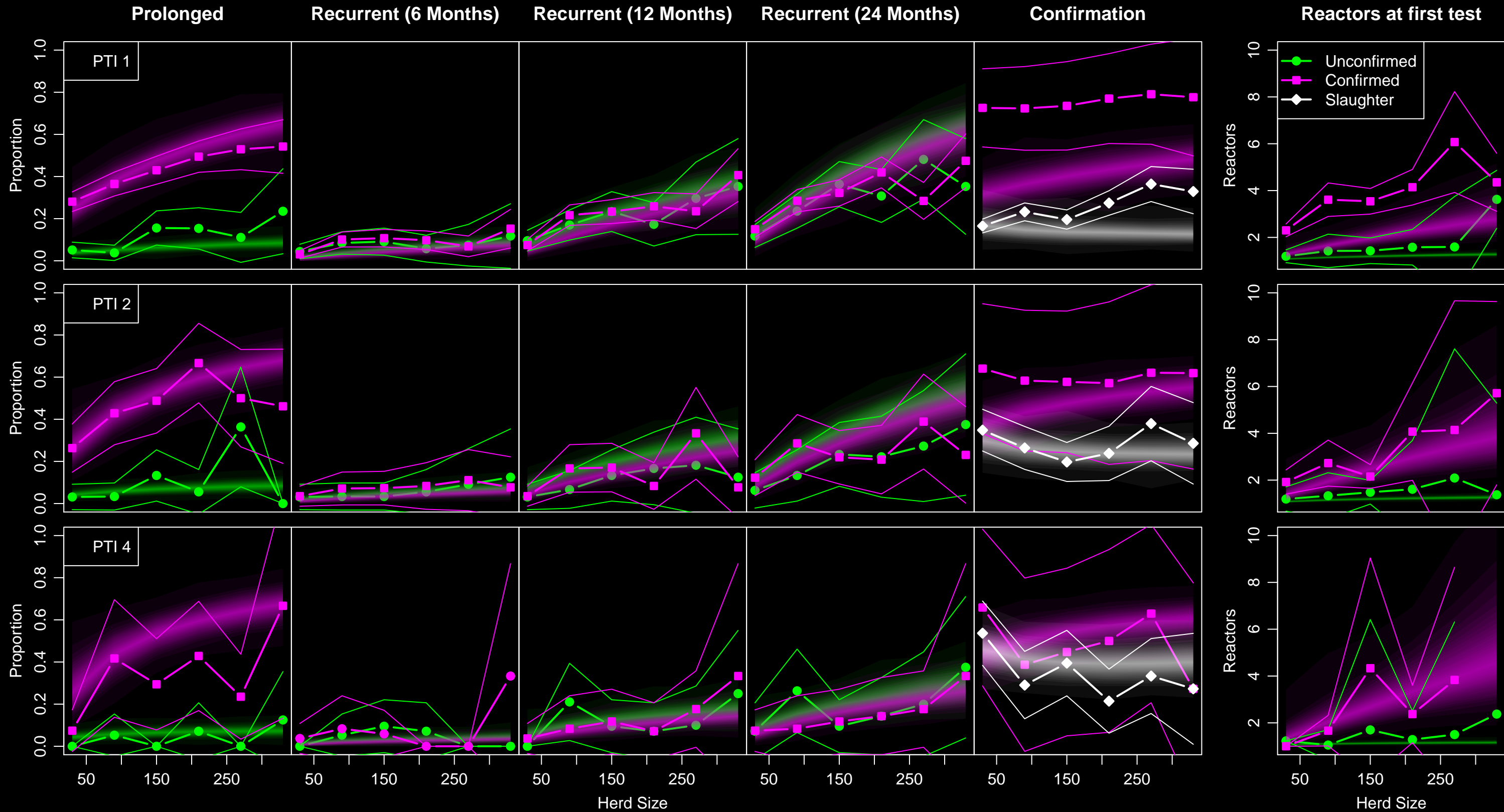

Supplement: Figure S7 — Target measures for the within-herd persistence of bTB (2006–2008) and predictive distributions for SORI model. The within-herd persistence of bTB in GB as measured by the probability of breakdowns being prolonged (duration of greater than 240 days) or recurrent within a 6, 12 and 24 month horizon. The relationship of each target measure is plotted against herd size, with breakdowns further stratified by parish testing interval (PTI 1 top row, PTI 2 middle row, PTI 4 bottom row) and confirmation status (confirmed breakdowns: green, circles, unconfirmed breakdowns: magenta squares). Target measures are calculated from breakdowns trigged within 2003–2005 by a routine surveillance test (VE-WHT, VE-WHT2, VE-RHT, VE-SLH). The probability of confirmation varies between PTI, as does the proportion of confirmed breakdowns initiated by a slaughterhouse case (white diamonds) and the mean number of reactors reported at the disclosing test. Uncertainty in each (mean) target observation (thick lines) is illustrated by an envelope (thin lines) of ±1.96 standard errors around the mean. Predictive distributions for each of these target measures from the finalized within-herd transmission model are plotted as shaded density strips where the intensity of color is proportional to the probability density at that point. (PDF) [file pcbi.1002730.s008.pdf]

**PTI 1****PTI 2****PTI 4**

Probability of Infection Remaining

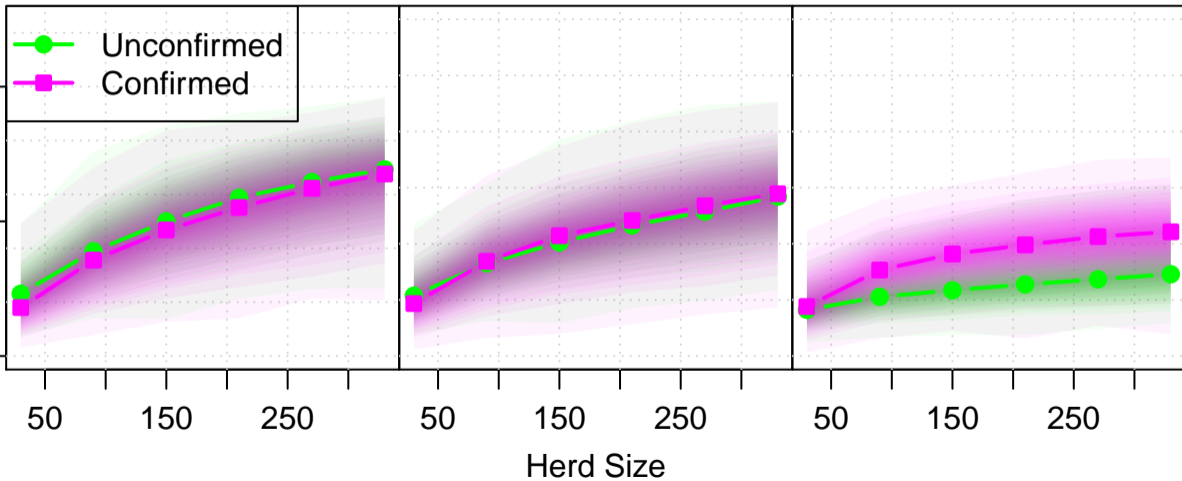

Supplement: Figure S8 — Burden remaining after resolution of a breakdown using SORI model. redictive distributions for the probability of at least one infectious bovine remaining within a herd after a breakdown is resolved as a function of herd size, classified by PTI (1,2,4 left to right) and confirmation status (Green circles confirmed, magenta squares unconfirmed). Predictive distributions are plotted as shaded density strips where the intensity of shading is proportional to the probability density at that point. Solid lines, and points for each herd-size category, indicate the median of the predictive distribution to aid comparison between confirmed and unconfirmed breakdowns. (PDF) [file pcbi.1002730.s009.pdf]

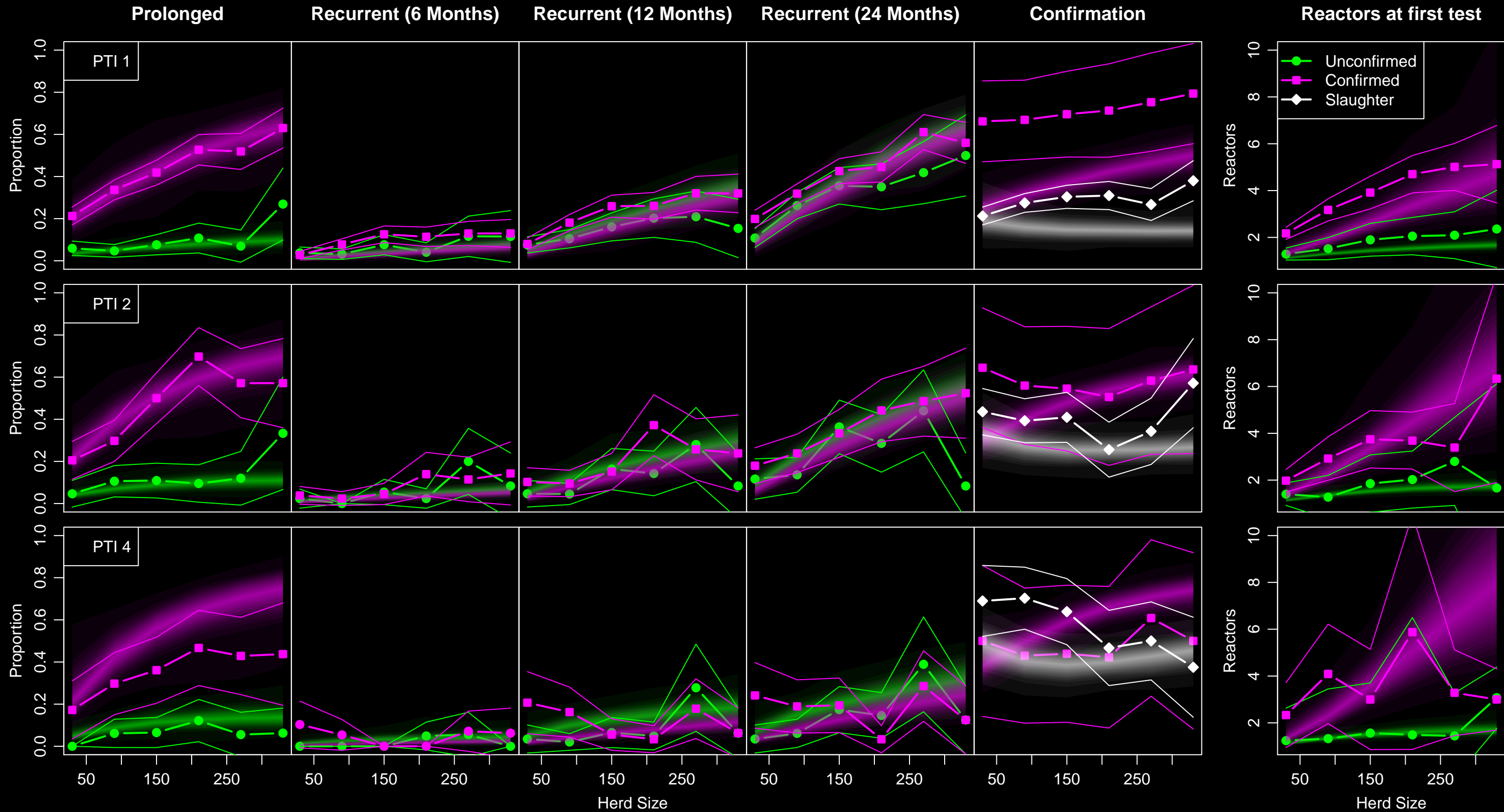

Supplement: Figure S10 — Target measures for the within-herd persistence of bTB (2003–2005) and predictive distributions for within-herd transmission model using SOR model. The within-herd persistence of bTB in GB as measured by the probability of breakdowns being prolonged (duration of greater than 240 days) or recurrent within a 6, 12 and 24 month horizon. The relationship of each target measure is plotted against herd size, with breakdowns further stratified by parish testing interval (PTI 1 top row, PTI 2 middle row, PTI 4 bottom row) and confirmation status (confirmed breakdowns: green, circles, unconfirmed breakdowns: magenta squares). Target measures are calculated from breakdowns trigged within 2003–2005 by a routine surveillance test (VE-WHT, VE-WHT2, VE-RHT, VE-SLH). The probability of confirmation varies between PTI, as does the proportion of confirmed breakdowns initiated by a slaughterhouse case (white diamonds) and the mean number of reactors reported at the disclosing test. Uncertainty in each (mean) target observation (thick lines) is illustrated by an envelope (thin lines) of ±1.96 standard errors around the mean. Predictive distributions for each of these target measures from the finalized within-herd transmission model are plotted as shaded density strips where the intensity of color is proportional to the probability density at that point. (PDF) [file pcbi.1002730.s011.pdf]

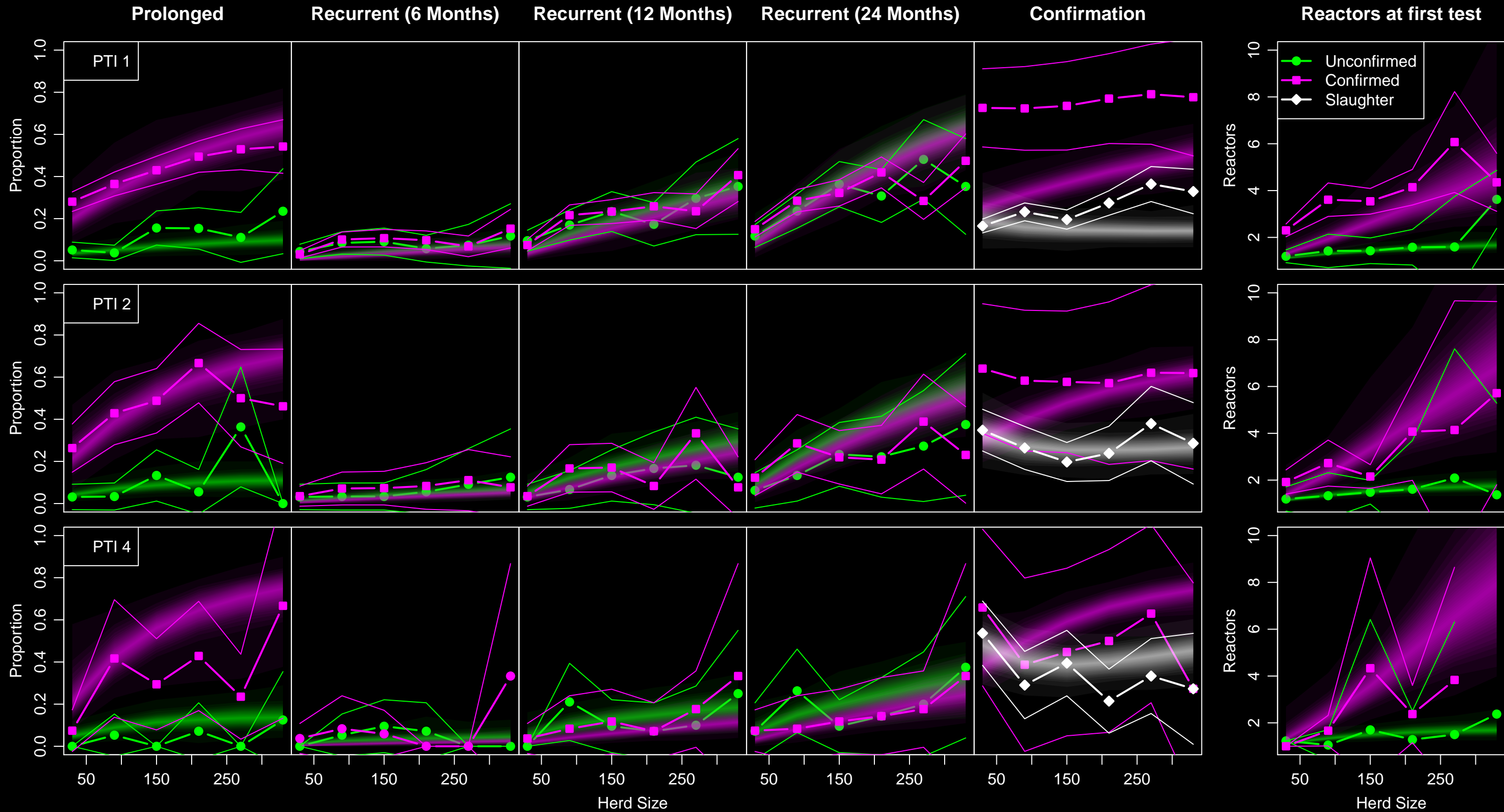

Supplement: Figure S11 — Target measures for the within-herd persistence of bTB (2006–2008) and predictive distributions for within-herd transmission model using SOR model. The within-herd persistence of bTB in GB as measured by the probability of breakdowns being prolonged (duration of greater than 240 days) or recurrent within a 6, 12 and 24 month horizon. The relationship of each target measure is plotted against herd size, with breakdowns further stratified by parish testing interval (PTI 1 top row, PTI 2 middle row, PTI 4 bottom row) and confirmation status (confirmed breakdowns: green, circles, unconfirmed breakdowns: magenta squares). Target measures are calculated from breakdowns trigged within 2003–2005 by a routine surveillance test (VE-WHT, VE-WHT2, VE-RHT, VE-SLH). The probability of confirmation varies between PTI, as does the proportion of confirmed breakdowns initiated by a slaughterhouse case (white diamonds) and the mean number of reactors reported at the disclosing test. Uncertainty in each (mean) target observation (thick lines) is illustrated by an envelope (thin lines) of ±1.96 standard errors around the mean. Predictive distributions for each of these target measures from the finalized within-herd transmission model are plotted as shaded density strips where the intensity of color is proportional to the probability density at that point. (PDF) [file pcbi.1002730.s012.pdf]

PTI 1

PTI 2

PTI 4

Probability of Infection Remaining

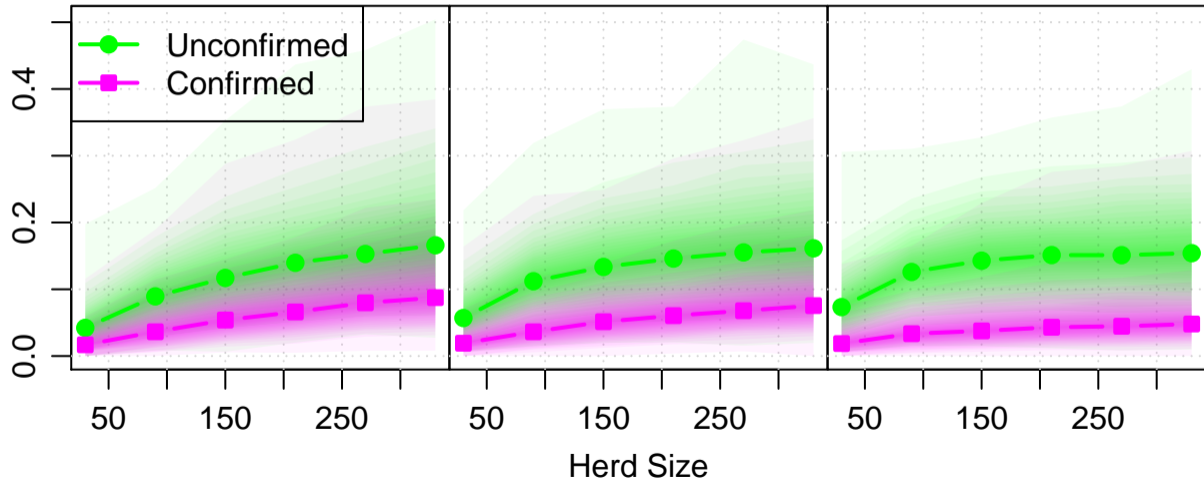

Supplement: Figure S12 — Burden remaining after resolution of a breakdown using SOR model. Predictive distributions for the probability of at least one infectious bovine remaining within a herd after a breakdown is resolved as a function of herd size, classified by PTI (1,2,4 left to right) and confirmation status (Green circles confirmed, magenta squares unconfirmed). Predictive distributions are plotted as shaded density strips where the intensity of shading is proportional to the probability density at that point. Solid lines, and points for each herd-size category indicate the median of predictive distribution to aid comparison between confirmed and unconfirmed breakdowns. (PDF) [file pcbi.1002730.s013.pdf]

Perfect Test

Perfect Isolation

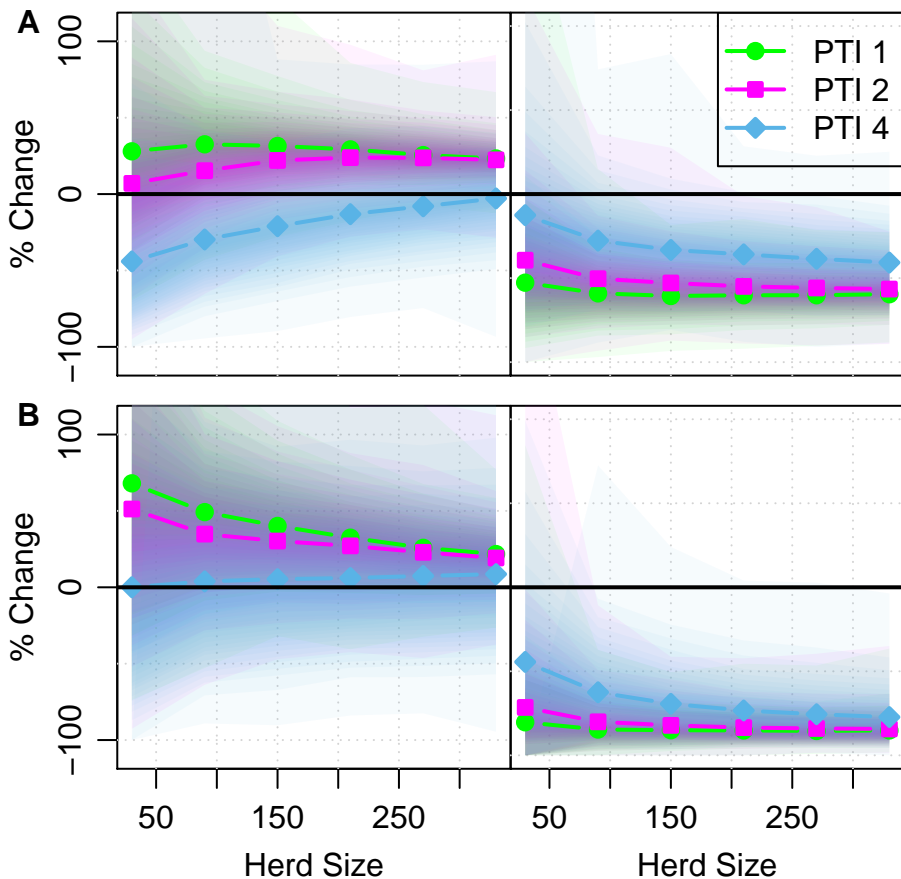

Supplement: Figure S13 — Impact of herd-level interventions on probability of recurrence within 24 months. Change in the probability of a herd experiencing a recurrent breakdown after application of a ‘perfect’ test (left column) or perfect isolation (right column). The perfect test is assumed to have 100% sensitivity and specificity and no occult period. Perfect isolation corresponds to setting the extrinsic infectious pressure to zero at the end of a breakdown (). Plotted values correspond to the average % difference in the probability of recurrence relative to the fitted SORI (Panel A) and SOR models (Panel B). Separate series are plotted for herds in PTI 1 (lime green circles), 2 (magenta squares) & 4 (sky blue diamonds). Predictive distributions are plotted as shaded density strips where the intensity of shading is proportional to the probability density at that point, with the mean of the predictive distribution plotted as a solid line. (PDF) [file pcbi.1002730.s014.pdf]
