# Supplementary material for: Estimating the Hidden Burden of Bovine Tuberculosis in Great Britain
Source: PLoS Comput Biol. 2012 Oct 18;8(10):e1002730. doi: 10.1371/journal.pcbi.1002730 (PMC3475695; doi:10.1371/journal.pcbi.1002730)
Supplement: Figure S5 — Estimated Parameter Distributions for SORI model. Distributions of parameters consistent with the persistence measures and reactor distributions estimated from VetNet data (Figure S6 ). The severe values of sensitivity and specificity are constrained to be greater than and less than their respective values at the standard interpretation. Likewise the probability of infected animals being detected by routine slaughterhouse surveillance is assumed to be less than or equal to the probability of confirmation. All parameters are constrained to be positive, with probabilities and the density dependent parameter q further constrained to be less than or equal to 1. (PDF) [file pcbi.1002730.s006.pdf]

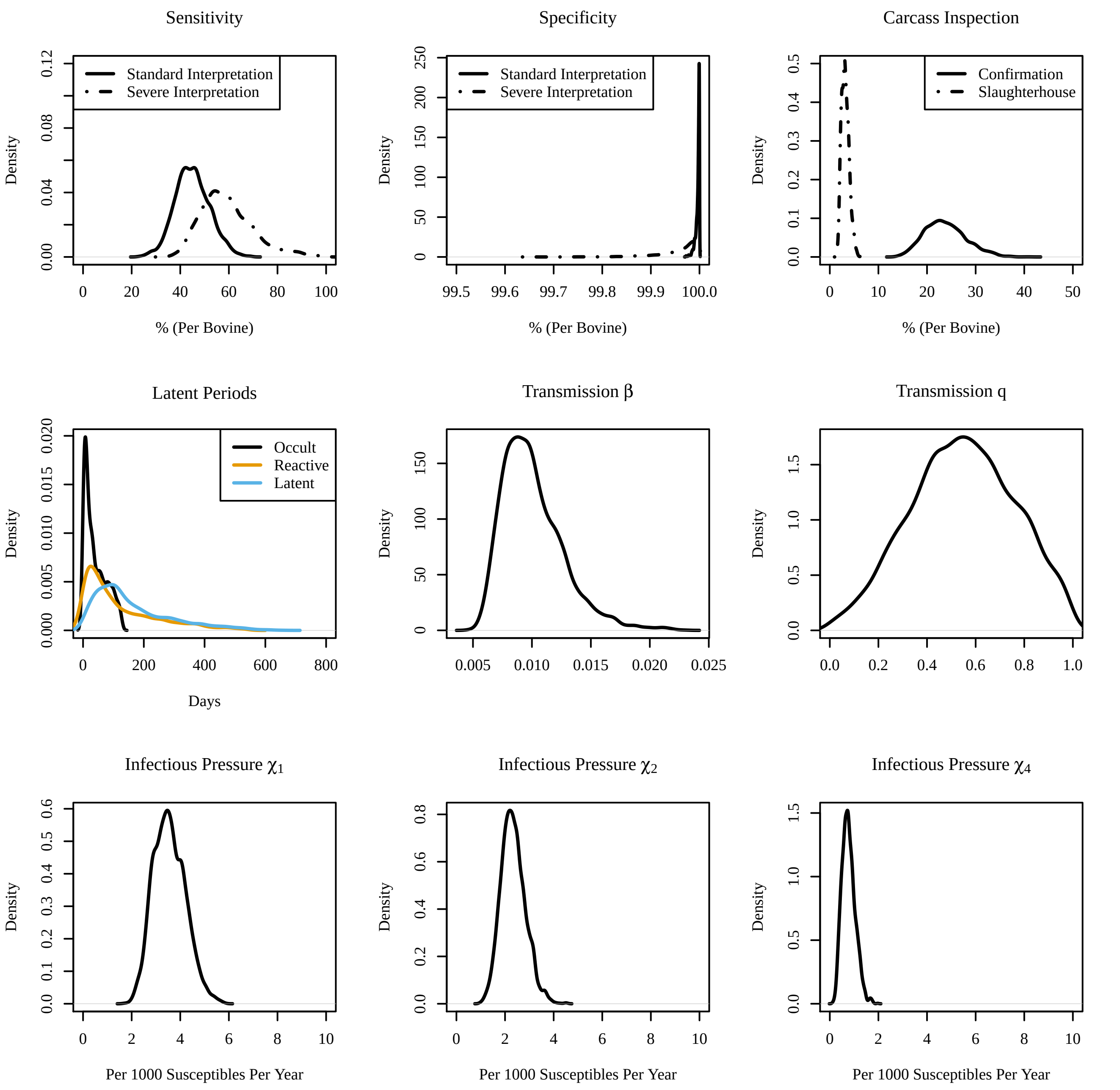
Sensitivity
Standard Interpretation
Severe Interpretation
Density
% (Per Bovine)
Specificity
Standard Interpretation
Severe Interpretation
Density
% (Per Bovine)
Carcass Inspection
Confirmation
Slaughterhouse
Density
% (Per Bovine)
Latent Periods
Occult
Reactive
Latent
Density
Days
Transmission β
Density
Transmission q
Density
Infectious Pressure χ_1
Density
Per 1000 Susceptibles Per Year
Infectious Pressure χ_2
Density
Per 1000 Susceptibles Per Year
Infectious Pressure χ_4
Density
Per 1000 Susceptibles Per Year
